# Context-dependent genetic architecture of Drosophila life span

**DOI:** 10.1371/journal.pbio.3000645

**Published:** 2020-03-05

**Authors:** Wen Huang, Terry Campbell, Mary Anna Carbone, W. Elizabeth Jones, Desiree Unselt, Robert R. H. Anholt, Trudy F. C. Mackay

**Affiliations:** Program in Genetics, W. M. Keck Center for Behavioral Biology and Department of Biological Sciences, North Carolina State University, Raleigh, North Carolina, United States of America; Institute of Science and Technology Austria (IST Austria), AUSTRIA

## Abstract

Understanding the genetic basis of variation in life span is a major challenge that is difficult to address in human populations. Evolutionary theory predicts that alleles affecting natural variation in life span will have properties that enable them to persist in populations at intermediate frequencies, such as late-life–specific deleterious effects, antagonistic pleiotropic effects on early and late-age fitness components, and/or sex- and environment-specific or antagonistic effects. Here, we quantified variation in life span in males and females reared in 3 thermal environments for the sequenced, inbred lines of the *Drosophila melanogaster* Genetic Reference Panel (DGRP) and an advanced intercross outbred population derived from a subset of DGRP lines. Quantitative genetic analyses of life span and the micro-environmental variance of life span in the DGRP revealed significant genetic variance for both traits within each sex and environment, as well as significant genotype-by-sex interaction (GSI) and genotype-by-environment interaction (GEI). Genome-wide association (GWA) mapping in both populations implicates over 2,000 candidate genes with sex- and environment-specific or antagonistic pleiotropic allelic effects. Over 1,000 of these genes are associated with variation in life span in other *D*. *melanogaster* populations. We functionally assessed the effects of 15 candidate genes using RNA interference (RNAi): all affected life span and/or micro-environmental variance of life span in at least one sex and environment and exhibited sex-and environment-specific effects. Our results implicate novel candidate genes affecting life span and suggest that variation for life span may be maintained by variable allelic effects in heterogeneous environments.

## Abstract 2

The effects of alleles associated with naturally occurring variation for lifespan in fruit flies are different in males and females and in different thermal environments. These genotype-by-sex and genotype-by-environment interactions may account for the maintenance of variation for lifespan in nature.

## Introduction

The human population is rapidly growing older [1,2], with a concomitant increase in age-related diseases [2,3] and societal challenges. Much of the recent increase in human life span can be attributed to improved sanitation and medical advances; however, there is also a genetic component. Estimates of heritability of human life span (the fraction of total variation in longevity attributable to genetic variation [4]) range from 0.10 to 0.30 [5–7], similar to heritability estimates for life span in other species [8]. Indeed, recent genome-wide association (GWA) studies of longevity in human populations have begun to elucidate some of the molecular variants associated with variation in life span, many of which are also associated with other quantitative traits and complex diseases [9–13]. The additive effects of the naturally occurring variants on the normal range of human longevity are individually small, as for other human quantitative traits, and therefore extremely large sample sizes are required for their detection [14]. Furthermore, the path from discovering a variant associated with a quantitative trait to validating a causal genotype–phenotype relationship is difficult in humans because of elevated levels of linkage disequilibrium (LD) in the human genome.

The maintenance of genetic variation for life span in natural populations is also an evolutionary conundrum. Some genetic variation in aging will be due to a balance between new deleterious mutations and their elimination by purifying natural selection; such alleles will be maintained at low equilibrium frequencies [4]. Evolutionary theories of aging that account for intermediate frequency alleles affecting life span invoke particular properties of these alleles. Medawar [15] recognized that the strength of natural selection declines with increasing age. He suggested that alleles with late-age–specific deleterious effects, after reproduction has ceased, will experience reduced natural selection and thus will be maintained at intermediate frequencies by mutation–selection balance (the “mutation accumulation” theory). Other theories postulate “antagonistic pleiotropic” effects of alleles affecting life span, for example, alleles with early-life beneficial fitness effects and late-life deleterious effects [16] or alleles with beneficial effects on reproduction but deleterious effects on somatic maintenance [17]. In addition, theories for the maintenance of quantitative genetic variation due to genotype-by-environment interaction (GEI) or genotype-by-sex interaction (GSI) also apply to life span. Alleles with environment- (or sex-)specific effects—or antagonistic effects in different environments or between the sexes—can be maintained at intermediate frequencies under some conditions [18–23].

Studies in short-lived model organisms, in particular *D*. *melanogaster*, have provided support for several of these evolutionary theories. Support for the mutation accumulation theory comes from observations of increased genetic variance in mortality [24,25] and fecundity [26] with increasing age. Support for the antagonistic pleiotropy theory comes from estimated negative genetic correlations between early fecundity and life span [27] and reduced early fecundity and increased life span of lines selected for late-age fecundity ([28–32] but see [33] and [34]). Additionally, single mutations affecting increased life span have deleterious effects on other fitness-related quantitative traits [35]. Many studies have reported GSI and GEI for natural variation in life span [36–43], but there are only a few examples in which candidate genes and variants have been mapped [44,45].

Identifying candidate genes and variants associated with naturally occurring variation for quantitative traits in *D*. *melanogaster* requires complete DNA sequences, because LD declines rapidly with physical distance in this species [46]. Here, we used the sequenced, inbred lines of the *D*. *melanogaster* Genetic Reference Panel (DGRP) [46,47] and an advanced intercross outbred population derived from a subset of DGRP lines [48] to address several questions regarding the genetic basis of naturally occurring variation for life span and the potential role of GSI and GEI in maintaining genetic variation for life span. (1) What genes harbor naturally occurring variants affecting life span? (2) To what extent are these genes the same as those identified from studies of induced mutations? (3) What is the magnitude of GSI and GEI? (4) What genes/variants are associated with GSI and GEI? (5) To what extent are GSI and GEI due to sex- and environment-specific allelic effects, and to antagonistic pleiotropic effects across sexes and environments? (6) To what extent are genes/variants associated with variation in life span shared in different populations? We observe that the genetic architecture of life span is highly context dependent and that the same genes are associated with variation in life span in different populations. We functionally assessed the effects of candidate genes using RNA interference (RNAi) and observed similar context-dependent effects on life span.

## Results

### Quantitative genetics of life span in the DGRP

**Fig 1:**
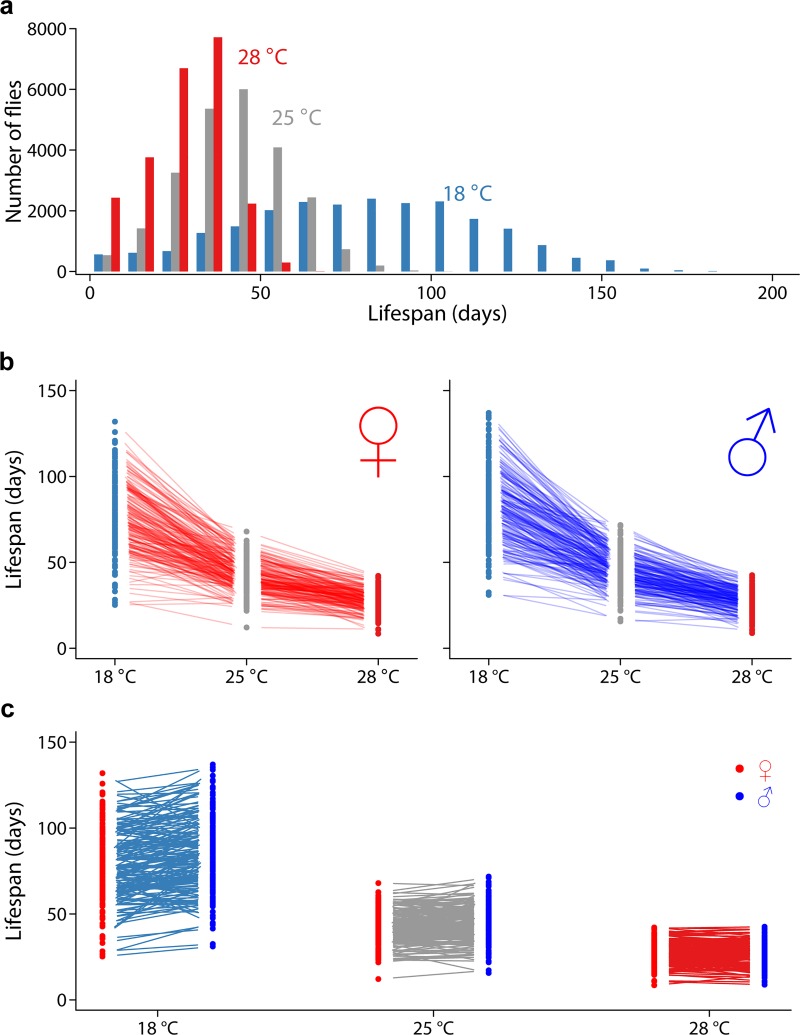
Overview of context-dependent genetic effects of life span in the DGRP. (A) Distribution of life span of individual flies across 3 temperatures. (B) Reaction norms of line means across 3 temperatures in females (left) and males (right). (C) Reaction norms of line means between the 2 sexes in each temperature. The raw data for the information depicted in this figure are available at https://github.com/qgg-lab/dgrp-lifespan/. DGRP, *D*. *melanogaster* Genetic Reference Panel.

We reared males and females from 186 DGRP lines in each of 3 thermal environments (18°C, 25°C, and 28°C) known to affect life span [39]. We measured the life span of a total of 70,209 flies (23,022 at 18°C, 24,060 at 25°C, and 23,127 at 18°C), controlling adult density by grouping 3 males and 3 females in each of 24 replicate vials per line and environment. We found substantial phenotypic variation in life span, ranging from 1 day to 188 days (Fig 1A). This replicated genotype design enables us to quantify the effects of genetic variation, sex, thermal macro-environments, and their interactions on phenotypic variation for life span.

Flies reared at 18°C lived on average twice as long as those reared at the standard laboratory temperature of 25°C, while those reared at 28°C had their average life span reduced by approximately 60% compared with that of flies reared under standard conditions (Fig 1A, Fig 1B, Table 1, S1 Table and S2 Table). At a population level, these statistics indicate that life span is highly plastic with respect to thermal rearing environment. There was significant genetic variance for life span in each thermal environment, with broad-sense heritabilities (*H*^2^) ranging from 0.36 to 0.42 (Table 1, S2 Table). The similar heritability (a unitless parameter of the ratio of genetic variance to total phenotypic variance) estimates disguise the fact that both genetic and environmental variances are greatly increased at 18°C and decreased at 28°C relative to 25°C (Table 1, S2 Table). On average, males lived significantly longer than females at 18°C and 25°C, but sexual dimorphism for life span was not significant at 28°C (Fig 1C, Table 1, S2 Table).

**Table 1:** Quantitative genetic parameters for life span and its micro-environmental variability in the DGRP.

Trait	Parameter	Temperature		
		18°C	25°C	28°C
**Life span**	$\bar{X}$ (female)	77.86	41.60	27.15
	$\bar{X}$ (male)	84.97	44.17	26.77
	$\sigma_{L}^{2}$	354.38	68.14	33.17
	$\sigma_{SL}^{2}$	84.98	28.77	9.12
	$\sigma_{G}^{2}$	439.36	96.91	42.29
	$\sigma_{\varepsilon }^{2}$	689.24	133.77	75.01
	$\sigma_{P}^{2}$	1,128.60	230.68	117.30
	*H*^2^	0.39	0.42	0.36
	*CV*_*G*_	25.75	22.96	24.12
	*CV*_*ε*_	32.25	26.97	32.12
**Life span micro-environmental variability**	$\bar{X}$ (female)	3.22	2.45	2.16
	$\bar{X}$ (male)	3.27	2.44	2.12
	$\sigma_{L}^{2}$	0.0531	0.0553	0.0622
	$\sigma_{SL}^{2}$	0.0210	0.0132	0.0186
	$\sigma_{G}^{2}$	0.0741	0.0685	0.0808
	$\sigma_{\varepsilon }^{2}$	0.0268	0.0354	0.0276
	$\sigma_{P}^{2}$	0.1009	0.1039	0.1084
	*H*^2^	0.73	0.66	0.74
	*CV*_*G*_	8.39	10.70	13.28
	*CV*_*ε*_	5.04	7.70	7.76

$\bar{X}$ (female, male): overall mean (female mean, male mean); $\sigma_{L}^{2}$: among-line variance component; $\sigma_{SL}^{2}$: sex-by-line interaction variance component; $\sigma_{G}^{2}$: total genetic variance $(\sigma_{L}^{2}+\sigma_{SL}^{2});\sigma_{\varepsilon }^{2}$: within-line variance; $\sigma_{P}^{2}$: total phenotypic variance ($\sigma_{G}^{2}+\sigma_{\varepsilon }^{2}$); *H*^2^: broad-sense heritability ($\sigma_{G}^{2}/\sigma_{P}^{2}$); *CV*_*G*_: coefficient of genetic variation ${(100\sigma }_{G}/\bar{X})$; *CV*_*G*_: coefficient of environmental variation ${(100\sigma }_{\varepsilon }/\bar{X})$. The variance components estimates are from the full model with each sex. The genetic components ($\sigma_{L}^{2}$ and $\sigma_{SL}^{2}$) are significant at *P* < 0.0001 in all analyses.

**Abbreviation:** DGRP, *D*. *melanogaster* Genetic Reference Panel

Life span in females and males was highly correlated within each thermal environment, with cross-sex genetic correlations of 0.81, 0.70, and 0.78 at 18°C, 25°C and 28°C, respectively. However, the sex-by-line interaction terms are significant for each temperature, indicating that these correlations are significantly less than unity and that there is genetic variation for sexual dimorphism of life span (i.e., GSI) in the DGRP (Table 2, S2 Table). We assessed to what extent the GSI is attributable to variation among the DGRP lines in the sign and magnitude of sex dimorphism for life span. Consistent with the similar level of genetic variance among sexes within each environment, the genetic variation in sex dimorphism in the DGRP is almost exclusively due to changes in rank order of life span as opposed to between-line variance for females and males within each temperature (Table 2), with extensive crossing of reaction norms for life span between males and females (Fig 1C).

**Table 2:** GSIs for life span and its micro-environmental variance (ln*σ*_*ε*_) in the DGRP.

Trait	Parameter		Temperature	
		18°C	25°C	28°C
**Life span**	*r*_*GMF*_	0.806	0.703	0.784
	*σ*_*LF*_	20.51	9.47	6.41
	*σ*_*LM*_	21.40	10.23	6.60
	*σ*_*LF*_*σ*_*LM*_(1−*r*_*GMF*_)	85.14	28.76	9.13
	(*σ*_*LF*_−*σ*_*LM*_)^2^/2	0.39	0.29	0.04
	% rank	99.5	99.0	99.6
**Life span micro-environmental variance**	*r*_*GMF*_	0.716	0.807	0.770
	*σ*_*LF*_	0.280	0.265	0.282
	*σ*_*LM*_	0.264	0.259	0.286
	*σ*_*LF*_*σ*_*LM*_(1−*r*_*GMF*_)	0.0210	0.0132	0.0185
	(*σ*_*LF*_−*σ*_*LM*_)^2^/2	0.0001	1.8 × 10^-5^	8.0 × 10^-6^
	% rank	99.5	99.9	100

*r*_*GMF*_: cross-sex genetic correlation ($\sigma_{L}^{2}/(\sigma_{L}^{2}+\sigma_{LS}^{2})$); *σ*_*LF*_: female genetic standard deviation; *σ*_*LM*_: male genetic standard deviation; % rank: contribution of changes in rank order of life span between males and females to sex-by-line interaction variance. All genetic components were significant at *P* < 0.0001.

**Abbreviations:** DGRP, *D*. *melanogaster* Genetic Reference Panel; GSI, genotype-by-sex interaction

The correlation of life span across thermal environments within each sex was quite low, however, ranging from 0.206 to 0.487 in females and 0.267 to 0.516 in males (Table 3). All temperature-by-line interaction terms (i.e., GEI) were highly significant for each pair of temperatures and across all temperatures (S2 Table). The GEI variance is mostly attributable to variation in the rank order of life span between 25°C and 28°C but to variation in both the rank order and between-line genetic variance of life span between 18°C and 25°C and 18°C and 28°C (Table 3). This is consistent with the markedly increased genetic variance for life span observed at 18°C and decreased genetic variance for life span observed at 28°C relative to 25°C. Across each pair of the 3 temperatures, approximately 60% of the GEI variance is attributable to changes in rank order among the DGRP lines in different temperatures, and the remaining approximately 40% is attributable to changes in among-line genetic variance with temperature.

**Table 3:** Genotype-by-temperature interactions for life span and its micro-environmental variance in the DGRP.

Trait	Sex	Parameter	Temperature comparison		
			18°C,25°C	18°C,28°C	25°C,28°C
****Life span****	****Female****	*r*_*GT*_	0.384	0.206	0.487
		*σ*_*Li*_	20.51	20.51	9.47
		*σ*_*Lj*_	9.47	6.41	6.41
		*σ*_*Li*_*σ*_*Lj*_(1−*r*_*GT*_)	119.65	104.37	31.14
		(*σ*_*Li*_−*σ*_*Lj*_)^2^/2	60.94	99.41	4.68
		% rank	66.3	51.2	86.9
	****Male****	*r*_*GT*_	0.442	0.267	0.516
		*σ*_*Li*_	21.40	21.40	10.23
		*σ*_*Lj*_	10.23	6.60	6.60
		*σ*_*Li*_*σ*_*Lj*_(1−*r*_*GT*_)	122.16	103.53	32.68
		(*σ*_*Li*_−*σ*_*Lj*_)^2^/2	62.38	109.52	6.59
		% rank	66.2	48.6	83.2
****Life span micro-environmental variability****	****Female****	*r*_*GT*_	0.493	0.182	0.495
		*σ*_*Li*_	0.280	0.280	0.265
		*σ*_*Lj*_	0.265	0.282	0.282
		*σ*_*Li*_*σ*_*Lj*_(1−*r*_*GT*_)	0.0376	0.0646	0.0377
		(*σ*_*Li*_−*σ*_*Lj*_)^2^/2	0.0001	2 × 10^−6^	0.0001
		% rank	99.7	100	99.7
	****Male****	*r*_*GT*_	0.326	0.087	0.589
		*σ*_*Li*_	0.264	0.264	0.259
		*σ*_*Lj*_	0.259	0.286	0.286
		*σ*_*Li*_*σ*_*Lj*_(1−*r*_*GT*_)	0.0461	0.0689	0.0304
		(*σ*_*Li*_−*σ*_*Lj*_)^2^/2	1.25 × 10^−5^	0.0002	0.0004
		% rank	100	99.7	98.7

*r*_*GT*_: cross-temperature genetic correlation ($\sigma_{L}^{2}/(\sigma_{L}^{2}+\sigma_{LT}^{2})$); *σ*_*Li*_, *σ*_*Lj*_: genetic standard deviations in temperatures *i* and *j*; % rank: contribution of changes in rank order of life span between temperatures to temperature-by-line interaction variance. All genetic components are significant at *P* < 0.0001.

**Abbreviation:** DGRP, *D*. *melanogaster* Genetic Reference Panel

Finally, when all life span data are considered across males and females and the 3 thermal environments, the cross-sex and environment genetic correlation (*r*_*GST*_) is only 0.31 (S2 Table). Genetic variation for life span is thus highly plastic, such that the phenotype of any one genotype varies substantially depending on sex and macro-environment (and the 3 environments assessed here are only a small fraction of environments the animals would encounter in nature). The low overall cross-sex and cross-environment genetic correlations indicate that the genetic basis of variation in life span within the same population is largely different depending on environmental and organismal context.

### Quantitative genetics of microenvironmental variance of life span in the DGRP

Because up to 72 individuals per sex for each of the DGRP lines had life span data in each of the 3 thermal macro-environments, we can also characterize genetic variation for within-line (micro-environmental) variance as well as its interaction with sex and thermal environment. Such micro-environmental variance may be due to the plasticity of life span in response to unmeasured environmental variation and intrinsic variability of each individual genotype, both of which may be under genetic control.

**Fig 2:**
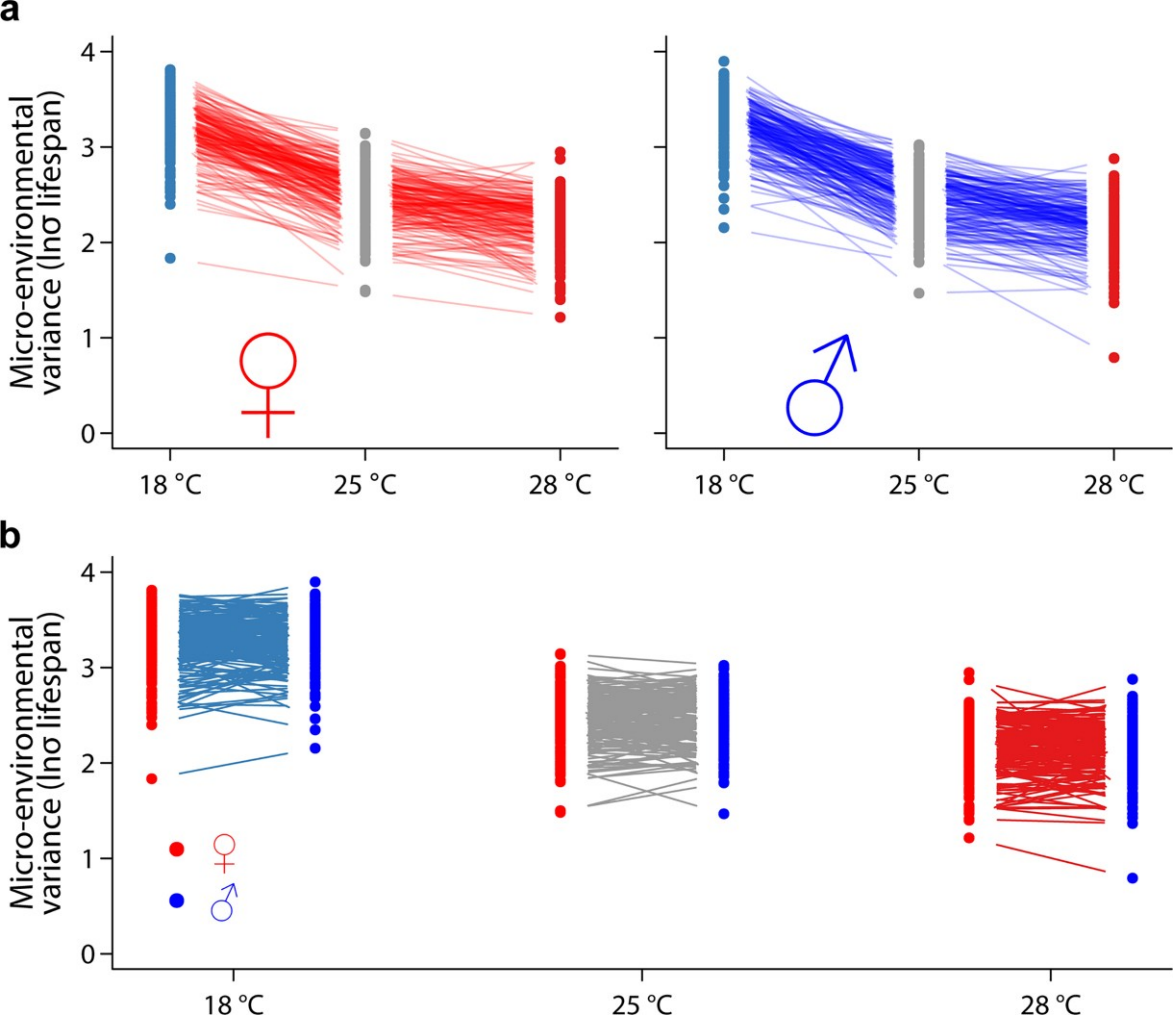
Overview of context-dependent genetic effects of micro-environmental variance of life span in the DGRP. (A) Reaction norms of ln*σ*_*ε*_ of life span across 3 temperatures in females (left) and males (right). (B) Reaction norms of ln*σ*_*ε*_ of life span between the 2 sexes in each temperature. The raw data for the information depicted in this figure are available at https://github.com/qgg-lab/dgrp-lifespan/. DGRP, *D*. *melanogaster* Genetic Reference Panel.

We first assessed whether there was heterogeneity of within-line micro-environmental variance between the lines and found highly significant differences in within-line variance of life span for all sex/temperature combinations. *P* < 0.0001 for Brown-Forsyth tests (Table 4) and Cochran’s C tests (Table 5) for all contexts. Moreover, similar to life span across the 3 thermal environments, natural log transformed within-line variance (ln*σ*_*ε*_) is increased at 18°C and decreased at 28°C relative to that of flies reared at the standard 25°C condition at a population level (Fig 2A, Table 2).

**Table 4:** Brown-Forsythe test for heterogeneity of within-line variances for life span.

****Sex****	****Environment****	****df****	****F****	*P* ****value****
****Females****	18°C	182, 11498	7.81	1.72 × 10^−177^
	25°C	185, 11969	7.60	3.73 × 10^−174^
	28°C	176, 11467	8.37	1.10 × 10^−188^
****Males****	18°C	182, 11158	5.77	4.02 × 10^−115^
	25°C	185, 11719	6.62	2.97 × 10^−143^
	28°C	176, 11306	7.20	7.23 × 10^−154^

**Table 5:** Cochran’s C tests for heterogeneity of within-line variances for life span.

****Sex****	****Environment****	****Average**** *n*	****k (lines)****	****C (test statistic)****	*P* ****value****
****Female****	18°C	64	183	0.0146	2.17 × 10^−9^
	25°C	65	186	0.0178	7.77 × 10^−16^
	28°C	66	177	0.0227	< 2.2 × 10^−16^
****Male****	18°C	62	183	0.0162	1.34 × 10^−11^
	25°C	64	186	0.0143	2.46 × 10^−9^
	28°C	65	177	0.0211	< 2.2 × 10^−16^

We require replication within each genotype to assess the magnitude of heritable micro-environmental variance in life span. However, each of the replicate vials only contains 3 individuals of each sex. We therefore randomly divided the 24 replicate vials into 2 groups within each line, sex, and thermal environment and computed the micro-environmental variance in each group. Similar to life span, micro-environmental variance also exhibits plasticity across temperature macro-environments (S3 Table). Broad-sense heritabilities of micro-environmental variability assessed across different DGRP lines are high, ranging from 0.66 to 0.74 (Table 1). Note, however, that these values are more akin to heritabilities of line means since the estimates are, by necessity, for groups of individuals and thus are greatly influenced by the number of individuals used to compute the variance. Although there was no significant difference in micro-environmental variance between males and females at 25°C averaged over all lines, there was significant sexual dimorphism for within-line variation of life span at 18°C and 28°C (S3 Table).

The cross-sex genetic correlations of ln*σ*_*ε*_ life span are high (0.72, 0.81, and 0.77 at 18°C, 25°C, and 28°C, respectively) but are all significantly different from unity, with significant GSI in each temperature environment. Similar to life span itself, variation for sex dimorphism of life span micro-environmental variance in the DGRP is almost exclusively due to changes in rank order within each temperature (Table 2), with extensive crossing of reaction norms for micro-environmental variance of life span between males and females (Fig 2B). The genetic correlation of micro-environmental variance of life span across temperatures is low, ranging from 0.18 to 0.50 in females and 0.09 to 0.59 in males. However, in this case the GEI variance is mostly attributable to variation in the rank order of ln*σ*_*ε*_ life span across temperatures (Table 3). The cross-sex and environment genetic correlation for ln*σ*_*ε*_ life span is *r*_*GST*_ = 0.32, and therefore the genetic basis of variation in micro-environmental plasticity is highly context dependent, like life span itself.

### GWA analyses in the DGRP

To gain insight into the genetic basis of variation for life span and the micro-environmental variance of life span, we performed GWA analyses to map variants associated with variation across DGRP lines in life span and ln*σ*_*e*_ of life span. Within each thermal environment, each GWA analysis evaluated the effects of common (minor allele frequency [MAF] > 0.05) variants on life span or ln*σ*_*e*_ life span for males, females, the average of males and females, and the difference between males and females (the variant-by-sex interaction effects) while accounting for any effects of *Wolbachia* infection, segregating polymorphic inversions, and polygenic relatedness [47]. We were able to obtain reliable phenotypes by averaging the life span for individual flies within each line for 183, 186, and 177 DGRP lines at 18°C, 25°C, and 28°C, respectively. We also performed similar analyses to evaluate the difference between thermal environments (the variant-by-temperature interaction effects) separately for males, females, and the average and difference of males and females. At a nominal *P* < 10^−5^, we identified a total of 1,105 variants affecting 659 genes (S4 Table). Quantile-quantile (QQ) plots of the *P* values indicated no obvious deviation from the expected uniform *P* value distribution when *P* values were large, whereas a small fraction of *P* values began to deviate from the expectation at the *P* value cut-off in most cases (S1 Fig). The majority of these variants are located in introns, upstream or downstream of the candidate genes, or in intergenic regions, suggesting that they may affect life span via modulation of gene expression. Except for a block of variants around the centromere of Chromosome 2, the variants were largely independent from each other (S2 Fig).

**Fig 3:**
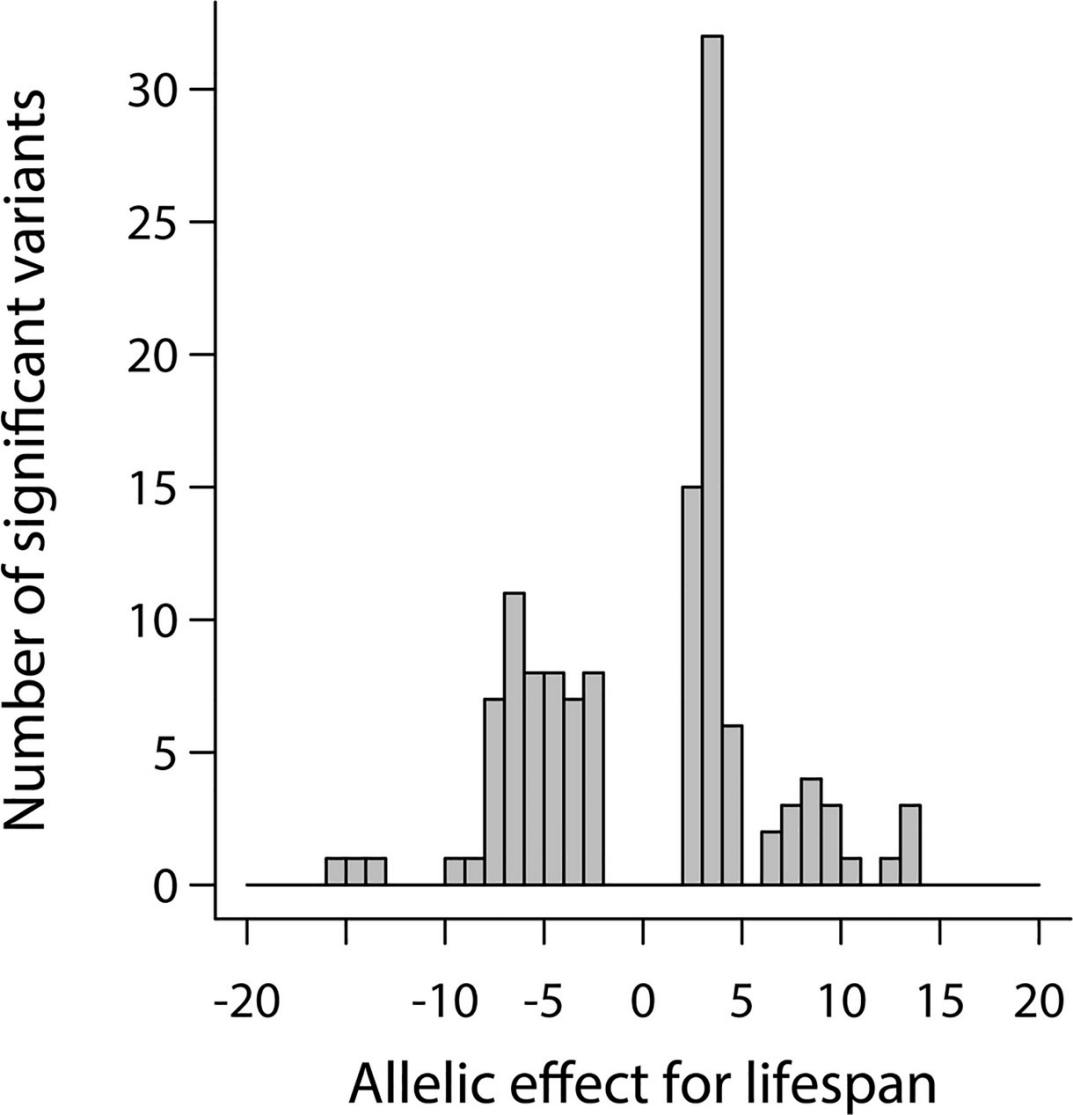
Distribution of allelic effects for life span in the DGRP. The raw data for the information depicted in this figure are available at https://github.com/qgg-lab/dgrp-lifespan/. DGRP, *D*. *melanogaster* Genetic Reference Panel.

Although the DGRP is underpowered to confidently map individual variants with small effects associated with life span and genetic variation in micro-environmental variability of life span between sexes and environments, we used the top associations as a group to evaluate the nature of genetic variation and GSI and GEI effects for life span and its micro-environmental variance. First, we note that many of the effects for life span within each sex and environment are negative; thus, it is the minor allele that is associated with increased longevity (Fig 3). Second, we do not find any overlap of the top variants affecting life span or ln*σ*_*ε*_ life span between sexes or temperatures, consistent with our inference from the quantitative genetic analyses that life span and ln*σ*_*ε*_life span are highly plastic traits with different genetic bases in different environments. Third, when effects of all variants on life span are considered, the Spearman correlation between the effect sizes in different environments is low, ranging from 0.33 to 0.51 when compared across temperatures and between 0.70 and 0.78 when compared between sexes in the same environment (S3 Fig). The same pattern was also observed for ln*σ*_*ε*_ (S4 Fig). Because the majority of variants have small effects, the correlation is driven by the few variants with larger effects and a general trend of the smaller effects. The relatively low correlation of effect sizes across environments suggests that the allelic effects are highly context dependent, consistent with the quantitative genetic analyses.

To understand the genetic basis of the sex/environmental plasticity of life span, we mapped variants that exhibited variant-by-sex and/or variant-by-temperature interaction. A variant-by-sex or variant-by-temperature interaction can arise if the sign of the allelic effect is the same across temperatures or sexes but differs in magnitude, or if the sign of the effect is in the opposite direction in males or females or across pairs of environments. The latter category of alleles exhibit antagonistic pleiotropy between sexes or environments. We identified 92 variants for which the *P* value of the difference between males and females for life span or ln*σ*_*ε*_ life span was <10^−5^ in any thermal environment (S4A Table). Remarkably, these variants had opposite effects in males and females, consistent with our inference from the quantitative genetic analyses that GSIs for life span and ln*σ*_*ε*_ life span within each environment are largely attributable to changes of rank order of effects between the sexes. We identified 279 variants for which the *P* value for the difference in life span or ln*σ*_*ε*_ life span was <10^−5^ in any pair of thermal environments for either males or females (S4B Table). Among these variants, the effects of 223 had different signs, while the remaining had the same sign but different magnitudes. Finally, we identified 114 variants for which the *P* value of the difference between males and females for the difference in life span or ln*σ*_*ε*_ life span between pairs of environments was <10^−5^ (S4C Table) (i.e., exhibit variant-by-sex-by-environment interaction). Most of these variants (110; 96.5%) had opposite effects in males and females.

### GWA analyses in the AIP

The advantage of the *Drosophila* system is that we can go beyond the restrictive sample size of the DGRP and map variants affecting life span with high confidence by generating an Advanced Intercross Population (AIP) from a subset of DGRP lines [48]. We maintained this population for over 100 generations with a large effective population size before performing “extreme quantitative trait locus (xQTL)” mapping [49] for life span. We aged a large (960 per sex) cohort of AIP individuals and sequenced whole genomes of pools of the longest living 10% (96) of individuals and randomly selected pools of the same number of young flies. We performed these analyses for males and females reared at each of the 3 temperatures and assessed whether there were significant differences in allele frequencies between the longest-lived and young pools. These variants are either the true causal variants or in LD with the true causal variants. We used *P* < 10^−7^ as a reporting threshold as variants with this level of significance exceed or are close to passing a stringent Bonferroni-corrected threshold for multiple testing (*P* = 5.08 × 10^−8^ for a 0.05 threshold). We identified 431, 614, and 248 variants in or near 271, 487, and 187 genes, respectively, for female life span at 18°C, 25°C, and 28°C and 145, 352, and 310 variants in or near 113, 252, and 212 genes, respectively, for male life span at 18°C, 25°C, and 28°C (S5A Table).

The 6 (2 sexes × 3 environments) xQTL GWA analyses were conducted using independent samples from the same AIP population. This allowed us to assess whether genetic effects replicate across sexes and environments, similar to the earlier analysis for the DGRP. Among the total of 2,074 variants that were associated with life span in at least one sex or environment, only 18 were identified in more than one experiment, including 12 in two experiments, 4 in three, and 2 in four (S5B Table). A total of 10 of these SNPs had similar effects in different experiments, but 8 SNPs had opposite effects between sexes and/or temperatures. The small degree of replication across experiments is not surprising given the significant GEI and GSI observed in the DGRP. On a global level, effects expressed as allele frequency difference between the long-living and randomly drawn pools had low correlation across environments, with correlation between the sexes at the same temperature the highest (S5 Fig).

**Fig 4:**
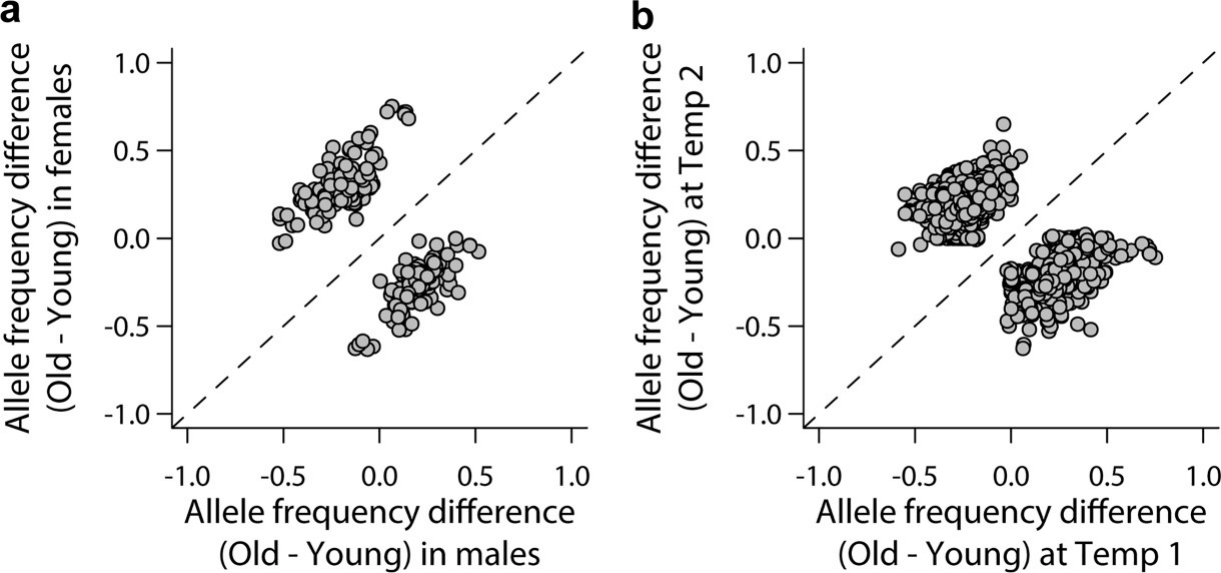
Antagonistic allelic effects for life span in the AIP. Scatter plots showing the comparison of allele frequency difference between the old and young pools in (A) one sex versus the other and (B) one temperature versus another. The raw data for the information depicted in this figure are available at https://github.com/qgg-lab/dgrp-lifespan/. AIP, Advanced Intercross Population.

To gain insights into the sex-specific and environment-specific genetic architectures, we mapped variants associated with the difference in life span between males and females within each thermal environment (GSI) and between pairs of environments for each sex (GEI). We identified 252 variants associated with the difference between males and females for all temperatures at *P* < 10^−7^ (S5C Table). A total of 113 variants were male- or female-specific (i.e., only significant in one sex), and 4 were significant in both sexes but with effects in opposite directions (S5B Table, S5C Table). The correlation of the difference in allele frequency between young and old samples in males and females was *r* = −0.61 (Fig 4A), again indicating substantial overall antagonistic pleiotropy between the sexes for variants associated with life span. Similarly, we identified 1,619 variants associated (*P* < 10^−7^) with the difference in life span between all pairs of temperatures for both sexes (S5D Table). A total of 581 variants were significant in only one temperature, and 6 variants had significant but opposite effects between temperatures (S5B and S5D Table). We also found pervasive antagonistic pleiotropic effects on life span across the different thermal environments: the correlation of the average difference in allele frequency between young and old samples between pairs of temperatures was *r* = −0.73 (Fig 4B).

In addition to single variants with opposite effects across sexes and temperatures, we also observe different SNPs within the same gene that have opposite effects in different conditions. A total of 49 genes have different SNPs with opposite effects in the same sex and environment, 29 genes have different SNPs with opposite effects for the same sex in different thermal environments, 18 genes have SNPs with opposite effects in males and females in the same environment, and 34 genes have SNPs with opposite effects in males and females in different environments (S5E Table). Thus, genes affecting life span have complex heterogeneous sex- and environment-dependent allelic effects, confirming our findings using the DGRP.

### Comparison between AIP and DGRP GWA analyses

While the sex- and environment-dependent allelic effects can explain the vastly different genes identified in the various analyses, they cannot explain the differences between the AIP and DGRP within a specific environment and sex. If we only consider the sex- and environment-specific analyses—i.e., analyses for one temperature and in one sex, and only life span (not micro-environmental variance)—no variant was in common among the 120 identified in the DGRP and among the 2,074 in the AIP. At the gene level, there were 23 genes that were common between the DGRP and AIP GWA analyses; however, the sex and/or temperature in which the genes were associated with life span in the 2 analyses were not always the same (S5F Table). There are several non–mutually exclusive explanations for this phenomenon: (1) The DGRP GWA analyses have low power. However, the large effect sizes implicated by the differences in allele frequency between long-lived and young flies for significant SNPs in the AIP analyses should be detectable in the DGRP GWA analyses. (2) Changes in LD between the DGRP and AIP mean that different variants in the same genes are associated with life span, or that LD extends further in the AIP than it does in the DGRP, implicating multiple variants across several genes in each LD block. (3) Variants have nonadditive effects—dominance, epistasis, or both—on life span. The DGRP lines are inbred, while the AIP is outbred: over- or underdominance of variants affecting life span would give different GWA results. However, the mean life span of the AIP population is very similar to that of the DGRP, rendering this unlikely. (4) In the presence of epistasis, differences in allele frequency of a focal variant between 2 populations will cause differences in the estimate of the effect of that variant between the populations [48,50,51]. Differences in allele frequency between the DGRP and AIP are expected since the AIP was derived from a subset of DGRP lines.

### Candidate genes associated with life span

We identified a total of 659 candidate genes associated with the different aspects of life span (life span, micro-environmental variance, inter-environmental, and cross-sex differences) in the DGRP GWA analyses and 1,857 candidate genes from the AIP GWA analyses (S6A Table). We performed Gene Ontology (GO) enrichment analyses [52–54] using the combined AIP and DGRP gene lists. If we do not account for gene length, we find enrichment (false discovery rate [FDR] < 0.05) for GO terms for aspects of nervous system development and function, general organismal growth and development, and behaviors. When we account for gene length differences and thus different baseline probabilities of being detected, no GO terms in the biological processes and cellular components categories were significant, and the most significant molecular function terms included serine-type endopeptidase inhibitor activity, L-ascorbic acid binding, and metalloendopeptidase activity (S6B Table).

Many genes affecting *Drosophila* life span are known from mutant and overexpression studies [35,55–62]. We evaluated to what extent 49 genes that affect life span when mutated or overexpressed are associated with naturally occurring variation in life span (S6C Table). A total of 16 genes (approximately 33%) overlapped this study and prior analyses of mutations/overexpression constructs affecting life span (*P* = 0.60 for enrichment). Given the context dependency and the different aspects of life span that we investigate in this study, this is not a surprising observation. This also illustrates the added power of harnessing natural variation.

Several other studies report GWA analyses for life span using full-sequence data. Two of these studies assessed mated [26] and virgin [63] DGRP flies. The other three studies [32,34,64] sequenced replicate lines maintained with generation intervals of 10 to 14 days (control lines) and replicate lines that were selected for postponed senescence, ultimately with generation intervals of 70 days [27,30,32]. These lines live up to twice as long as the control lines [34]. We performed pairwise comparisons of gene overlap between each of these studies (S7A Table) and the genes implicated in this study and identified 1,008 genes that were common to at least 2 analyses (S7B Table). These independently replicated genes are thus top candidates for further functional analyses. They are enriched for GO terms involving development and morphogenesis, in particular development and function of the nervous system, and behaviors in the analyses not corrected for gene length; however, no GO terms were significantly enriched following gene length adjustment (S7C Table). A total of 663 of these genes had at least one human ortholog (S7D Table).

### Functional assessment of candidate genes

As noted earlier, the majority of variants in candidate genes associated with life span are noncoding and presumably regulatory. We used RNAi [65] using a weak ubiquitous *GAL4* driver line (*Ubi156-GAL4*) [66] to assess whether reducing gene expression for a sample of candidate genes affected life span and whether these effects were also sex specific and thermal environment specific. We chose 15 candidate genes for which the RNAi construct was inserted in the same genomic location and evaluated the life span of males and females at 18°C, 25°C and 28°C. A total of 9 of these candidate genes (*bru-3*, *CG11828*, *CG13921*, *Cht-2*, *Doa*, *Eip75B*, *PGRP-LA*, *PGRP-LC*, and *Rdl*) were implicated in this study and that of Carnes and colleagues [34], 8 of them (*bru-3*, *CG42750*, *Doa*, *E23*, *Eip75B*, *olf413*, *Rdl*, and *tou*) were identified in several analyses in this study, and 2 of them (*CG9265* and *Cka*) were identified only in the 25°C analysis of the difference between males and females in the DGRP.

**Fig 5:**
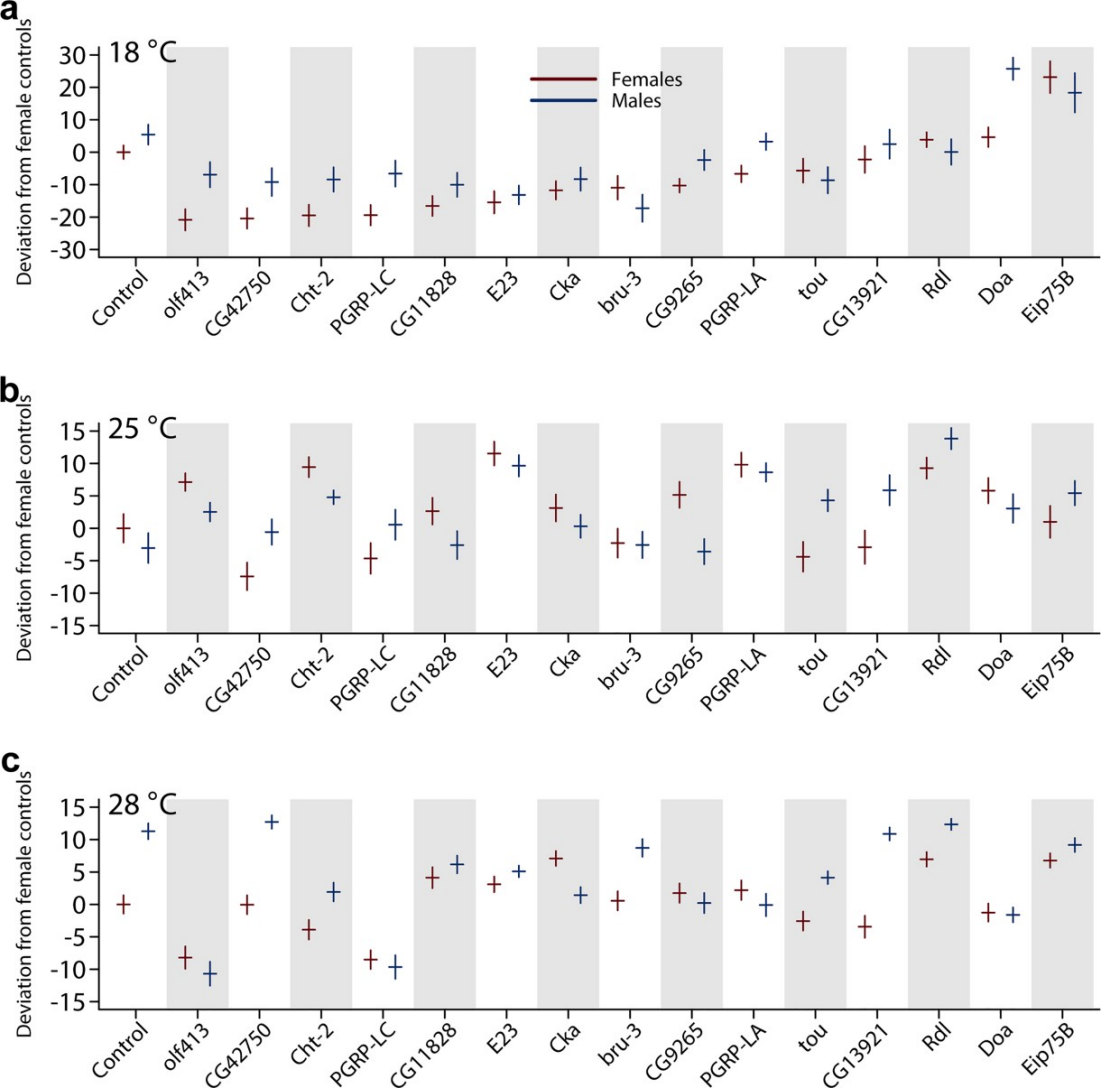
RNAi functional assessments of candidate genes. Mean (horizontal lines) ± one standard error (vertical lines) of life span of RNAi genotypes and their controls in each temperature. Life span is expressed as the deviation from the mean of the female control flies in each temperature such that the relative magnitude of life span is retained across temperatures. The genes on the *x* axis are ordered based on the mean of female life span at 18°C. Red = females and blue = males. (A) 18°C; (B) 25°C; (C) 28°C. *P* values of differences between sexes, genotypes, and temperatures are given in S8 Table. The raw data for the information depicted in this figure are available at https://github.com/qgg-lab/dgrp-lifespan/. RNAi, RNA interference.

We used an ANOVA to partition the variation in life span between the main effect of control and RNAi genotypes, thermal environments, and males and females, and the two-and three-way interactions (S8 Table, Fig 5). Although the micro-environmental variance was high, all candidate genes tested had significant effects on life span in at least one sex and environment, all but *CG13921* had significant temperature-by-genotype interactions, 13 of the candidate genes had significant GSIs, and for 8 of the candidate genes, the magnitude of the difference between the sexes between the control and RNAi genotype was different at different temperatures (i.e., the temperature-by-sex-by-genotype interaction was significant) (S8Q Table). Among the genes with significant genotype-by-temperature interactions, 6 had environment-specific effects, while 8 had opposite effects in at least 2 environments. Only 2 of the genes with genotype-by-sex effects had opposite effects in males and females; the others had sex-specific effects. The significant effects of RNAi knockdown of gene expression on 9 genes in males and/or females at 25°C led to increased life span relative to the co-isogenic control in all but one instance (S8Q Table), suggesting that reduction in gene expression is not unconditionally deleterious at this temperature. In contrast, RNAi mostly led to decreased life span at 18°C and 28°C, with a few exceptions (*Doa* males and *Eip75B* males and females at 18°C and *CG11828*, *Cka*, *Eip75B*, and *Rdl* females at 28°C) (S8Q Table, Fig 5).

We also assessed to what extent RNAi knockdown of gene expression affected within-genotype micro-environmental variance of life span. A total of 11 of the candidate genes exhibited differences in within-genotype variance compared to the control in at least one sex and temperature at *P* < 0.01 (S9 Table, Fig 5). In addition, we performed mixed-model ANOVAs to evaluate to what extent within-line variability of life span—parameterized as ln*σ*_*ε*_—between the RNAi lines and their co-isogenic controls had sex- and temperature-specific effects, and we observed pervasive context-dependent effects (S10 Table).

In summary, all RNAi constructs in candidate genes implicated by the GWA analyses affect life span in at least one sex and environment, validating the GWA results at the level of candidate genes. The effects of RNAi constructs on life span depend on sex and thermal environment, as observed for naturally occurring allelic variants, and RNAi constructs can also affect within-line variance for life span in a context-dependent manner.

## Discussion

We quantified male and female life span for inbred, sequenced DGRP lines and sequenced pools of young and old flies from an AIP derived from a subset of DGRP lines, in each of 3 thermal environments. We used these data to perform a quantitative genetic analysis of life span, map candidate genes and variants affecting life span, determine the extent to which alleles affecting life span have context-dependent effects, and infer evolutionary mechanisms maintaining naturally segregating variation for life span. To our knowledge, this is the largest genetic analysis of life span to date performed in a model system.

Our quantitative genetic analysis of variation in life span in the DGRP revealed considerable genetic variation in each thermal environment, with broad-sense heritabilities of *H*^2^ ~ 0.40. There is substantial GSI and GEI for life span. At a global level, GSI is largely due to changes in rank order of life span between males and females, indicating that antagonistic pleiotropy is responsible for genetic variation in sexual dimorphism for life span. GEI is partially attributable to changes in rank order between environments and partly to changes in variance between environments, indicating that antagonistic pleiotropy between environments as well as environment-specific effects is responsible for genetic variation in plasticity of life span between environments.

The different DGRP genotypes also displayed significant variation in the magnitude of within-line variation (micro-environmental plasticity [67]) for life span, with broad-sense heritabilities even higher than those for life span (*H*^2^ = 0.71, on average). The study of genetic variation of micro-environmental variation is in its infancy, but the phenomenon appears to be common as it has been demonstrated previously for *Drosophila* bristle number [68], sleep traits and waking activity [69], starvation resistance, chill coma recovery time and locomotor startle response [70], left-right turn bias [71], and food consumption [66]. Notably, genetic control of micro-environmental plasticity is specific for each trait [70]. Here, we demonstrate that there is GSI and GEI for micro-environmental plasticity of life span, due to antagonistic pleiotropy between sexes and between thermal environments.

We mapped genes and variants associated with variation in mean life span and micro-environmental variance of life span in the DGRP—as well as genes and variants associated with changes in allele frequency between young and old flies in the AIP—separately for males and females in each thermal environment. In addition, we mapped genes and variants associated with GSI and GEI in both populations. The vast majority of variants associated with life span in each context (2 sexes and 3 environments) were specific to one context. Although only a few polymorphic variants had significant individual antagonistic pleiotropic effects on life span in males and females or across pairs of environments, overall the effects of variants associated with GSI and GEI were negatively correlated, indicating substantial antagonistic pleiotropy consistent with the quantitative genetic analyses. Note that this is antagonistic pleiotropy for alleles associated with life span itself, and not between life span and another fitness trait [16]. However, we note that it is often the minor allele that is associated with increased life span. This strongly suggests that these alleles negatively impact fitness via other traits and are maintained segregating via antagonistic pleiotropy *sensu* Williams [16].

The net result of pervasive context-dependence effects of alleles associated with life span is that variants associated with life span under one condition will have dramatically diminished effects when averaged over all other conditions. Thus, genetic variation for life span might be maintained by variation in the magnitude and direction of natural selection due to context-specific allelic effects in heterogeneous environments as well as by antagonistic pleiotropic effects of variants on life span or on other fitness traits in different contexts. In addition to single variants with context-dependent effects, we also observe different variants in the same gene, often in close physical proximity, that have opposite effects depending on sex or environment. Thus, net effects of variants within a gene can cancel out due to complex heterogeneous sex- and environment-dependent allelic effects. Human GWA analyses for quantitative traits and common diseases in very large populations typically identify variants with very small effects associated with these phenotypes [72]. Perhaps these small effects are also the result of averaging over many different environmental conditions and/or heterogeneous effects of variants within a gene or LD block. Accounting for different environmental exposures and full genome sequencing may reveal context-dependent variation in effect size and local genetic heterogeneity.

If natural variation in life span is maintained by GSI and GEI for life span itself and antagonistic pleiotropy with other fitness traits, and if there are genes in which mutations with such effects tend to occur, then we would predict that there would be significant overlap in genes associated with increased life span in different populations. It is—perhaps remarkably—easy to select for increased life span by progressively delaying the age of reproduction [29,30,32]. Each of these laboratory evolution experiments was derived from a different United States population of flies: Michigan [30], Massachusetts [29], and New Jersey [32]. The DGRP lines were derived from a North Carolina population in 2003 [46]. Comparison of these data sets gives an unprecedented opportunity to inquire whether particular genes are more likely to harbor variants associated with life span. Therefore, we extended a previous analysis [64] comparing the overlap of genes harboring the top variants reported from pooled DNA sequencing analyses of these lines [32,34,64] to include the 3 DGRP-based analyses (this study; [26,63]) and identified 1,008 genes shared between at least 2 studies. The overlap between this study and those of Remolina and colleagues, Carnes and colleagues, and Fabian and colleagues [32,34,64] is quite remarkable since the studies span over 3 decades, have different experimental designs, use different reporting *P* value significance thresholds, and use different geographic populations. In addition, the gene lists in all cases are inflated by single variants between genes or that could affect overlapping genes; all associated genes are reported. The laboratory evolution studies will have different LD structures than the DGRP due to smaller sample sizes and selection, also increasing the number of reported genes.

The 1,008 genes replicated among these studies are top candidates for further functional analysis. Since 66% of these genes have human orthologs, they are candidates for “public” mechanisms of aging, shared among taxa [73]. They include genes affecting nervous system development and function and could potentially contribute to diseases associated with the cognitive decline and locomotor impairment associated with ageing. Most of the variants associated with variation in life span are in putative regulatory regions that may affect variation in transcript abundance. RNAi knockdown of gene expression is an imperfect proxy for the effects of naturally occurring variants because we cannot mimic the magnitude, timing, and tissue specificity of transcriptional variation possibly caused by naturally occurring variants. Nevertheless, the 15 RNAi constructs examined in both sexes and in the same 3 environments affected life span in at least one environment and sex. In addition, 12 of the RNAi constructs affected micro-environmental plasticity of life span in at least one sex and thermal environment. Thus, different molecular perturbations of these candidate genes recapitulate the context-dependent effects of naturally occurring variants. In the future, precise allelic replacement of candidate variants using CRISPR/*Cas-9* gene editing will be needed to confirm causal associations.

## Materials and methods

### *Drosophila* stocks

We used 186 of the inbred, sequenced DGRP lines [46,47] and an AIP derived from 40 randomly selected DGRP lines [48] that were maintained by random mating at large census population size (400 males and females per generation) for over 100 generations prior to the beginning of the assays described subsequently. We also used 15 KK (*bru-3*, *CG11828*, *CG13921*, *CG42750*, *CG9265*, *Cht2*, *Cka*, *Doa*, *E23*, *Eip75B*, *Ino80*, *olf413*, *PGRP-LA*, *PGRP-LC*, *Rdl*, *tou*) *UAS*-RNAi lines [65], their respective co-isogenic control stock, *y*,*w*^1118^;*P*{*attP*,*y*^+^,*w*^3^} (VIE-260B, Vienna stock 60100), and a weak ubiquitous *GAL4* driver (*Ubi156-GAL4* [66]) derived in our laboratory. The weaker *Ubi156-GAL4* driver line was used to prevent lethality often observed with stronger ubiquitin promoters. All stocks were routinely maintained at 25°C on cornmeal-molasses-agar medium (cornmeal, 65 g/L; molasses, 45 ml/L; yeast, 13 g/L) under a 12:12 hour light:dark cycle.

### Life span assays

We performed life span assays in each of 3 thermal environments: 18°C, 25°C, and 28°C. To minimize larval density effects, all experimental flies were produced for each genotype by allowing 6 males and 6 females to mate and lay eggs for 1 day in vials containing 10 ml culture medium, in each thermal environment. Offspring from these vials were collected at 1 to 3 days post eclosion for life span assays. We could not obtain sufficient life span data for 3 lines at 18°C and 9 lines at 28°C due to poor viability at these temperatures.

Life span was assessed for the DGRP lines in each temperature using 24 replicate vials per line and temperature containing 5 ml culture medium. We placed three 1- to 3-day-old males and three 1- to 3-day-old females in each replicate vial, for a total of 72 flies/sex/line/temperature. We transferred the flies without anesthesia to new vials containing 5 ml of fresh food and recorded deaths every 2 to 3 days, until all flies were dead.

We reared the AIP lines at the same 3 temperatures. For each temperature, we collected 2 replicates of 96 five-day-old mated males and females, and we froze the 12 pools of 96 flies (3 temperatures, 2 replicates/temperature, 2 sexes) at −80°C. We also collected 1,920 one- to three-day-old males and females at each temperature and placed 3 male and 3 female AIP flies in each of 640 vials, which we split into 2 replicates of 320 vials each. We transferred the flies to new vials every 2 to 3 days until 96 flies/sex/replicate remained alive, and we froze the 12 pools of flies at −80°C.

We crossed virgin females from the *UAS*-RNAi lines to *Ubi156-GAL4* males and measured life span as described for the DGRP, but increasing the sample size to 48 replicate vials with 3 males and 3 females per genotype. The controls for these crosses were F1 offspring from crosses of co-isogenic *w*^1118^ VIE-260B virgin females to *Ubi156-GAL4* males.

### Quantitative genetics of life span in the DGRP

We partitioned variation in life span in the DGRP into components attributable to the main cross-classified effect of sex, thermal environment, and DGRP line and their two- and three-way interactions using a mixed-model factorial ANOVA: *Y* = *μ* + *L* + *S* + *T* + *L*x*S* + *L*x*T* + *S*x*T* + *L*x*S*x*T* + *Rep*(*L*x*T*) + *S*x*Rep*(*L*x*T*) +*ε*, in which *Y* is the phenotype, *μ* is the overall mean; *S* and *T* are the fixed effects of sex and thermal environment, respectively; *L* is DGRP line (random); *L*x*S*, *L*x*T*, *S*x*T*, and *L*x*S*x*T* are the interaction terms; *Rep* is replicate (random); and *ε* is the error variance. We estimated the broad-sense heritability (*H*^2^) of life span from the full model as *H*^2^ = $\left(\sigma_{L}^{2}+\sigma_{LS}^{2}+\sigma_{LT}^{2}+\sigma_{LST}^{2})/(\sigma_{L}^{2}+\sigma_{LS}^{2}+\sigma_{LT}^{2}+\sigma_{LST}^{2}+\sigma_{\varepsilon }^{2}\right)$ in which $\sigma_{L}^{2},\sigma_{LS}^{2},\sigma_{LT}^{2},\sigma_{LST}^{2}$, and $\sigma_{\varepsilon }^{2}$ are the among-line, line x sex, line x age, line x sex x age, and within-line variance components, respectively.

We also ran reduced ANOVA models pooled across temperatures, separately for males and females; pooled across sexes, separately for each temperature; and within each temperature and sex. We estimated cross-sex genetic correlations separately for each temperature as *r*_*GMF*_ = $\sigma_{L}^{2}/(\sigma_{L}^{2}+\sigma_{LS}^{2})$, cross-temperature genetic correlations separately for males and females as *r*_*GT*_ = $\sigma_{L}^{2}/(\sigma_{L}^{2}+\sigma_{LT}^{2})$, and the overall genetic correlation across sexes and temperatures as *r*_*GST*_ = $\sigma_{L}^{2}/(\sigma_{L}^{2}+\sigma_{LS}^{2}+\sigma_{LT}^{2}+\sigma_{LST}^{2})$. We estimated the extent to which the *L*x*S* variance components are due to variation among the DGRP lines in the sign and magnitude of the difference in life span between males and females as $\sigma_{LS}^{2}=\sigma_{LF}\sigma_{LM}(1-r_{GFM})+{\left(\sigma_{LF}-\sigma_{LM}\right)}^{2}/2$, in which *σ*_*LF*_ and *σ*_*LM*_ are the square roots of the among-line variance components for females and males, respectively [74]. We estimated the extent to which the *L*x*T* variance components are due to variation among the DGRP lines in the sign and magnitude of life span differences between temperatures as $\sigma_{LT}^{2}=\sum \left[2\sigma_{Li}\sigma_{Lj}(1-r_{ij})+{\left(\sigma_{Li}-\sigma_{Lj}\right)}^{2}]/t(t-1\right)$, in which *t* = 3 temperatures, *σ*_*Li*_ and *σ*_*Lj*_ are the square roots of the among-line variance components for temperatures *i* and *j*, and *r*_*ij*_ is the cross-temperature correlation among lines for temperatures *i* and *j* [74]. The first term in these expressions reflects the amount of interaction variance caused by changes in the rank order of lines between sexes or thermal environments, while the second reflects the amount of interaction variance caused by differences in the magnitude of among-line variance between sexes or temperatures.

In addition to assessing genetic variation in life span in the 3 thermal environments, we assessed the extent to which environmental variance (i.e., variation within lines, or micro-environmental plasticity) was under genetic control. First, we tested for heterogeneity of within-line variances among lines, separately for males and females within each temperature, using the Brown-Forsythe test and Cochran’s C test. The Brown-Forsythe test uses a transformation of the response variable as $z_{ij}=|y_{ij}-\bar{y_{j}}|$, in which *y*_*ij*_ is the *i*^th^ individual of the *j*^th^ line and $\bar{y_{j}}$ is the median of the *j*^th^ line and significance of variance heterogeneity is tested using ANOVA of the transformed variable. Cochran’s C test computes the test statistic $C_{j}=\frac{S_{j}^{2}}{\sum_{i=1}^{N}S_{i}^{2}}$, in which $S_{j}^{2}$ is the variance within the line with the largest variance whereas $S_{i}^{2}$ is the variance within each line. Next, we pooled individual life span data from replicate vials 1–12 as “Replicate 1” and vials 13–24 as “Replicate 2” for the purpose of estimating quantitative genetic parameters for micro-environmental plasticity. We estimated the variance of life span within Replicate 1 and Replicate 2 separately for each line, sex, and environment and used ln*σ*_*ε*_ as our metric of micro-environmental variation, in which *σ*_*ε*_ is the square root of the within-line variance estimates [70,75,76]. Note that our estimates of the within-line variance include both the variance between replicate vials as well as the variance within replicate vials; both components of variance reflect the micro-environmental plasticity. We partitioned variation in ln*σ*_*ε*_ among lines, temperatures, and sexes using cross-classified mixed-effect factorial ANOVAs and their reduced models exactly as described earlier for life span, with the exception that there were no terms including “*Rep*.” We also computed heritabilities, cross-sex genetic correlations, and cross-environment genetic correlations, as described for life span.

#### GWA analyses in the DGRP

We performed GWA analyses for DGRP line means and mean ln*σ*_*ε*_ within each thermal environment. The analyses evaluated the strength of association for each of 1,890,367 polymorphic variants for which the MAF was >0.05. Single-marker analyses were performed separately for males and females and for the average and difference between the two sexes while accounting for any effects of *Wolbachia* infection, common polymorphic inversions, and polygenic relatedness, as described previously [47]. The difference between the two sexes models the sex-by-line interaction effects. We also performed GWA analyses for line means and mean ln*σ*_*ε*_ within each sex but considering all 3 pairs of thermal environments (18°C and 25°C, 25°C and 28°C, and 18°C and 28°C) separately for males and females. These analyses specifically evaluated the strength of association for the difference in life span between thermal environments, which models the GEI effect. Finally, we performed GWA analyses for line means and mean ln*σ*_*ε*_ for the differences between pairs of thermal environments, separately for males and females and for the average and difference between the two sexes. The difference between males and females in these analyses represents the sex-by-temperature-by-line interaction effect.

#### GWA analyses in the AIP

We sequenced DNA from each of the 24 pools of randomly collected and longest living AIP flies from each replicate, sex, and temperature to 130–156× coverage per pool and assessed allele frequency differences between the long-lived and control pools of individuals. We extracted genomic DNA from each of the 24 samples of 96 flies and used high–molecular-weight double-stranded genomic DNA samples to construct Illumina paired-end libraries. Briefly, 300 ng of DNA in a total volume of 50 μl was fragmented using ultra-sonication (Covaris, Woburn, MA) to an average size of 300–400 bp. We used 50 ng of the fragmented DNA for library preparation as follows. Fragments were purified with 1.8X Agencourt AMPure XP beads (Beckman-Coulter, Brea, CA) and then subjected to end-repair (Enzymatics, now Qiagen, Hilden, Germany), adenylation of 3′-ends (Enzymatics), and ligation of indexed paired-end adapters (Enzymatics and Bioo Scientific, Austin, TX). Each step was followed by purification using 1.8X Agencourt AMPure XP beads (Beckman-Coulter). After ligation, size selection was carried out with 0.5X PEG/NaCl and purification with 0.1X Agencourt AMPure XP beads. PCR enrichment of the purified barcoded DNA was carried out with KAPA HiFi Hot Start Mix (Kapa Biosystems, https://www.sigmaaldrich.com/life-science/roche-biochemical-reagents/kapa-genomics-reagents.html) and NEXTflex Primer Mix (Bioo Scientific). The libraries were quantified using the Quant-iT dsDNA high-sensitivity assay kit (Invitrogen, Carlsbad, CA). The sizes of the PCR-enriched libraries were quantified with an Agilent 2100 Bioanalyzer using the high-sensitivity DNA chip (Agilent, Santa Clara, CA). We divided the 24 libraries into 3 multiplexed pools of 8 libraries and sequenced each of the multiplexed pools on each of 3 HiSeq2500 (Illumina, San Diego, CA) lanes (i.e., 9 lanes total) with v4 chemistry. Clonal clusters were generated on an Illumina C-Bot with Illumina’s paired-end flow cell; 2 × 125 bp cycles of sequencing with 7 cycles of index sequencing were carried out according to the manufacturer’s standard protocol (Illumina). Imaging analysis and base calling were carried out with RTA software on the HiSeq2500. CASAVA 1.7 was used to demultiplex the sequences into fastq files that were used in the mapping analysis.

We mapped sequence reads to the *D*. *melanogaster* reference genome (FlyBase version 5.57 of the BDGP5 assembly) using BWA-MEM [77] and subsequently indel realigned, duplicate masked, and quality recalibrated using GATK [78]. Alleles at segregating sites in the 40 AIP parental lines were counted in the top (H) and random (C) pools, and their frequencies were compared (*f*_*H*_ − *f*_*C*_) using a *Z* test [48]. To obtain comparable tests to the GWA analyses in the DGRP that mapped variants contributing the sex-by-line and temperature-by-line interaction, we also tested differences in *f*_*H*_ − *f*_*C*_ across sexes within thermal environments and across all pairs of thermal environments with sexes using *Z* tests [79]. All sequence data can be accessed through the Sequence Read Archive with accession number PRJNA577841. All phenotype data as well as codes to analyze them are provided at https://github.com/qgg-lab/dgrp-lifespan/.

#### GO enrichment

We performed standard GO enrichment analyses as well as GO enrichment analyses accounting for differences in gene length [52–54]. Briefly, we randomly drew the same number of genes without replacement with probability proportional to lengths of the genes in the original set. The randomly drawn genes were then intersected with each GO term, and the null distribution of the intersection was obtained by performing the random sampling 10,000 times. The *P* value of the enrichment was calculated as (*B* + 1)/(*N* + 1), in which *B* is the number of samplings in which the enrichment was greater than or equal to the observed enrichment and *N* is the total number of samplings (10,000).

#### Functional assessment of candidate genes

We tested whether 15 candidate genes implicated from the GWA analyses affected life span when gene expression was knocked down using RNAi [65] and a weak ubiquitous RNAi driver (*Ubi156-GAL4*) derived in our laboratory [66]. We crossed the RNAi lines and their co-isogenic control line to *Ubi156-GAL4* and quantified the life span of RNAi and control F1 genotypes at all 3 temperatures. We note that the RNAi and control lines have been maintained independently for 10 years and that they may have diverged with respect to life span due to accumulation of spontaneous mutations affecting life span. However, the same RNAi and control genotypes for each gene were tested for effects on life span at 3 temperatures and in both sexes—variation in differences between these genotypes in different temperatures and sexes cannot be explained by differences in genetic background. We partitioned variation in life span using the mixed-model factorial ANOVA: *Y* = *μ* + *G* + *S* + *T* + *G*x*S* + *G*x*T* + *S*x*T* + *G*x*S*x*T* + *Rep*(*G*x*T*) + *S*x*Rep*(*G*x*T*) + *ε*, in which *G* is the main effect of genotype (RNAi or control, fixed), *S* is the main effect of sex (fixed), *T* is the main effect of temperature (fixed), and *Rep* is replicate (random). We also performed reduced analyses by sex and temperature. We tested whether RNAi affected heterogeneity of within-line variance, separately for males and females within each temperature, using Levene tests, as described earlier for the DGRP lines. We also performed the quantitative genetic analyses of micro-environmental plasticity as described for the DGRP lines, in which replicates 1–24 were pooled as “Replicate 1” and Replicates 25–48 were pooled as “Replicate 2” within each genotype, sex, and environment.

## Supporting information

S1 Table Mean life span (days) and micro-environmental variance (lnσε) in the DGRP. NA, not applicable due to poor viability. (XLSX)

S2 Table ANOVAs of life span measured at 25°C, 18°C, and 28°C. Sex, temperature, and their interaction are fixed effects; the rest are random. Full mixed model, factorial ANOVAs as well as reduced models by temperature and sex are given. *σ*^2^, REML variance component estimate; df, degrees of freedom; F, F-ratio test; *H*^2^, broad-sense heritability; *L*, DGRP Line; MS, Type III mean squares; *Rep*, Replicate vial; *S*, Sex; SE, standard error; *T*, Temperature.
(DOCX)

S3 Table ANOVAs of life span micro-environmental variance (ln*σ*_*ε*_) measured at 25°C, 18°C, and 28°C. Sex, temperature, and their interaction are fixed effects; the rest are random. Full mixed model, factorial ANOVAs as well as reduced models by temperature and sex are given. *σ*^2^, REML variance component estimate; df, degrees of freedom; F, F-ratio test; *H*^2^, broad-sense heritability; *L*, DGRP Line; MS, Type III mean squares; *Rep*, Replicate vial; *S*, Sex; SE, standard error; *T*, Temperature.
(DOCX)

S4 Table GWA analyses for mean life span and ln*σ*_*ε*_ life span in the DGRP. (A) GWA analyses within each temperature for females, males, the average of the two sexes, and the difference between the sexes. (B) GWA analyses within each sex for pairs of temperatures including each temperature individually and their average and difference. (C) GWA for the difference between temperatures for females, males, the average of the two sexes, and the difference between the sexes.
(XLSX)

S5 Table GWA analyses of life span in the AIP. (A) GWA within each temperature/sex combination. (B) SNPs in common between the different GWA analyses. (C) GWA analysis for the difference between females and males in each temperature. (D) GWA analysis for the difference between pairs of temperatures in each sex. (E) Genes found in common between the different GWA analyses in the AIP. (F) Genes found in common between the GWA analyses in the AIP and DGRP. SNP, single nucleotide polymorphism.
(XLSX)

S6 Table Gene-level analyses. (A) Genes discovered by GWA analyses in the AIP and DGRP and their union. (B) GO enrichment analyses. (C) Comparison of genes discovered in this study and genes with mutations affecting life span.
(XLSX)

S7 Table Comparison of life span candidate genes from published GWA analyses. (A) Candidate genes from previous GWA analyses of life span in the DGRP and in laboratory evolution lines selected for postponed reproductive senescence. (B) Candidate genes that overlap between at least two studies. (C) GO enrichment analyses of candidate genes that overlap between at least two studies. (D) Human orthologs of candidate genes that overlap between at least two studies. Only genes with DIOPT scores of 3 or greater are listed. DIOPT, *D**rosophila* RNAi Screening Center Integrative Ortholog Prediction Tool.
(XLSX)

S8 Table RNAi functional assessments of candidate genes. (A) Summary statistics of line means and standard errors (SEs) in each RNAi and control genotype. (B–P) Full-model and reduced-model ANOVAs for each gene. (Q) Summary of *P* values.
(XLSX)

S9 Table Tests for variance heterogeneity in RNAi experiments. (A) Estimates of within-line variance for each genotype/sex/temperature. (B) Summary of *P* values for the variance heterogeneity tests.
(XLSX)

S10 Table Quantitative genetic analyses for micro-environmental variance of RNAi and control genotypes of candidate genes. (A) ANOVA for micro-environmental variance within each temperature/sex combination. (B–P) Full-model and reduced-model ANOVAs for micro-environmental variance for each gene. (Q) Summary of *P* values from the ANOVA.
(XLSX)

S1 Fig QQ plots of *P* values for GWA studies in the DGRP. QQ plots of *P* values where the *x* axis is the expected *P* value based on a uniform distribution while the *y* axis is the observed *P* value. The horizontal line indicates the 10^−5^ cutoff chosen to declare significance. Code to generate the Q-Q plots is available at https://github.com/qgg-lab/dgrp-lifespan/.
(TIFF)

S2 Fig LD between significant variants in the DGRP. Heatmap showing the pairwise *r*^2^ between significant variants in the DGRP. Below the heatmap, the relative positions between the variants are indicated on the chromosomes by lines connecting the positions on the heatmap and positions on the chromosomes. The raw data for the information depicted in this figure are available at https://github.com/qgg-lab/dgrp-lifespan/.
(TIFF)

S3 Fig Context-dependent allelic effects for life span in the DGRP. Estimated allelic effects (for mean life span) in each environment are plotted against each other where the *x* axis is the effect in the environment indicated on the top of the column of cells (along the diagonal) and the *y* axis is the effect in the environment indicated on the right of the row of cells (along the diagonal). The data are plotted as a smoothed two-dimensional density plot with large effects (low-density areas) plotted as points on the edge. The darkness of the color indicates density of points. Spearman’s correlation is also indicated on the top left corner of the plot. The raw data for the information depicted in this figure are available at https://github.com/qgg-lab/dgrp-lifespan/.
(TIF)

S4 Fig Context-dependent allelic effects for life span in the DGRP. Estimated allelic effects (for ln*σ*_*ε*_ life span) in each environment are plotted against each other where the *x* axis is the effect in the environment indicated on the top of the column of cells (along the diagonal) and the *y* axis is the effect in the environment indicated on the right of the row of cells (along the diagonal). The data are plotted as a smoothed two-dimensional density plot with large effects (low-density areas) plotted as points on the edge. The darkness of the color indicates density of points. Spearman’s correlation is also indicated on the top left corner of the plot. The raw data for the information depicted in this figure are available at https://github.com/qgg-lab/dgrp-lifespan/.
(TIF)

S5 Fig Context-dependent allelic effects for life span in the AIP. Estimated allelic effects (allele frequency difference between the long-living and random pools) in each environment are plotted against each other where the *x* axis is the effect in the environment indicated on the top of the column of cells (along the diagonal) and the *y* axis is the effect in the environment indicated on the right of the row of cells (along the diagonal). The data are plotted as a smoothed two-dimensional density plot with large effects (low-density areas) plotted as points on the edge. The darkness of the color indicates density of points. Spearman’s correlation is also indicated on the top left corner of the plot. The raw data for the information depicted in this figure are available at https://github.com/qgg-lab/dgrp-lifespan/.
(TIF)

## Abbreviations

AIP: Advanced Intercross Population
DGRP: *D*. *melanogaster* Genetic Reference Panel
FDR: false discovery rate
GEI: genotype-by-environment interaction
GO: Gene Ontology
GSI: genotype-by-sex interaction
GWA: genome-wide association
LD: linkage disequilibrium
MAF: minor allele frequency
QQ: quantile-quantile
RNAi: RNA interference
xQTL: extreme quantitative trait locus
