# Supplementary material for: Context-dependent genetic architecture of Drosophila life span
Source: PLoS Biol. 2020 Mar 5;18(3):e3000645. doi: 10.1371/journal.pbio.3000645 (PMC7077879; doi:10.1371/journal.pbio.3000645)
Supplement: S2 Table — Sex, temperature, and their interaction are fixed effects; the rest are random. Full mixed model, factorial ANOVAs as well as reduced models by temperature and sex are given. σ2, REML variance component estimate; df, degrees of freedom; F, F-ratio test; H2, broad-sense heritability; L, DGRP Line; MS, Type III mean squares; Rep, Replicate vial; S, Sex; SE, standard error; T, Temperature. (DOCX) [file pbio.3000645.s002.docx]

| **Analysis** | **Source** | **df** | **MS** | **F** | ***P*** | ***σ*^2^ (SE)** | ***H*^2^** |  |
| --- | --- | --- | --- | --- | --- | --- | --- | --- |
| Full Model | *S* | 1 | 170,039 | 42.22 | 7.23e-10 | Fixed | 0.40 |  |
|  | *T* | 2 | 17,553,262 | 1,265.00 | 4.82e-163 | Fixed |  |  |
|  | *S*×*T* | 2 | 81,554 | 33.62 | 4.16e-14 | Fixed |  |  |
|  | *L* | 185 | 37,773 | 2.44 | 1.87e-13 | 59.78 (11.15) |  |  |
|  | *S*×*L* | 185 | 4042 | 1.67 | 2.22e-5 | 8.72 (2.50) |  | |
|  | *T*×*L* | 358 | 13,914 | 5.53 | 7.16e-56 | 93.01 (8.57) |  | |
|  | *S*×*T*×*L* | 358 | 2432 | 6.25 | 2.76e-241 | 32.58 (2.90) |  | |
|  | *Rep*(*T*×*L*) | 12,057 | 478 | 1.22 | 6.62e-29 | 16.35 (1.45) |  | |
|  | *S*×*Rep*(*T*×*L*) | 12,057 | 390 | 1.33 | 8.67e-92 | 32.38 (1.96) |  | |
|  | Error | 45,003 | 293 |  |  | 295.73 (1.98) |  | |
| Females (all temperatures) | *T* | 2 | 7,739,751 | 936.14 | 5.86e-143 | Fixed | 0.39 | |
|  | *L* | 185 | 19,563 | 2.36 | 1.95e-12 | 59.98 (11.45) |  | |
|  | *T*×*L* | 358 | 8,976 | 19.32 | 0 | 125.06 (9.85) |  | |
|  | *Rep*(*T*×*L*) | 12,057 | 432 | 1.52 | 1.37e-157 | 50.21 (2.22) |  | |
|  | Error | 22,877 | 285 |  |  | 287.42 (2.70) |  | |
| Males (all temperatures) | *T* | 2 | 9,864,990 | 1,226.60 | 5.81e-161 | Fixed | 0.40 | |
|  | *L* | 185 | 22,230 | 2.76 | 1.06e-16 | 77.12 (13.18) |  | |
|  | *T*×*L* | 358 | 8,070 | 18.56 | 0 | 125.95 (9.93) |  | |
|  | *Rep*(*T*×*L*) | 12,057 | 438 | 1.45 | 2.77e-125 | 47.34 (2.32) |  | |
|  | Error | 22,126 | 301 |  |  | 304.10 (2.90) |  | |
| 18°, 25° | *S* | 1 | 275,927 | 55.45 | 3.50e-12 | Fixed | 0.40 | |
|  | *T* | 1 | 17,107,358 | 984.45 | 2.42e-75 | Fixed |  | |
|  | *S*×*T* | 1 | 62,384 | 19.94 | 1.40e-5 | Fixed |  | |
|  | *L* | 185 | 42,845 | 2.22 | 2.01e-8 | 94.74 (19.58) |  | |
|  | *S*×*L* | 185 | 4,990 | 1.59 | 8.82e-4 | 15.47 (4.91) |  | |
|  | *T*×*L* | 182 | 17,418 | 5.35 | 1.50e-28 | 115.08 (14.93) |  | |
|  | *S*×*T*×*L* | 182 | 3135 | 5.95 | 3.10e-118 | 41.19 (5.16) |  | |
|  | *Rep*(*T*×*L*) | 8,138 | 651 | 1.23 | 3.77e-21 | 22.92 (2.41) |  | |
|  | *S*×*Rep*(*T*×*L*) | 8,138 | 529 | 1.32 | 3.78e-58 | 43.33 (3.26) |  | |
|  | Error | 30,068 | 401 |  |  | 404.50 (3.31) |  | |
|  |  |  |  |  |  |  |  | |
|  |  |  |  |  |  |  |  | |
| Females  (18°, 25°) | *T* | 1 | 7,671,303 | 736.79 | 6.68e-66 | Fixed | 0.39 | |
|  | *L* | 185 | 22,641 | 2.17 | 1.18e-7 | 97.49 (20.63) |  | |
|  | *T*×*L* | 182 | 10,439 | 18.00 | 0 | 156.16 17.26) |  | |
|  | *Rep*(*T*×*L*) | 8,138 | 584 | 1.51 | 4.06e-103 | 67.44 (3.68) |  | |
|  | Error | 15,329 | 388 |  |  | 390.95 (4.48) |  | |
| Males  (18°, 25°) | *T* | 1 | 9,470,415 | 938.63 | 9.17e-74 | Fixed | 0.40 | |
|  | *L* | 185 | 25,169 | 2.49 | 7.13e-10 | 123.00 (23.09) |  | |
|  | *T*×*L* | 182 | 10,120 | 17.01 | 0 | 156.19 (17.32) |  | |
|  | *Rep*(*T*×*L*) | 8,138 | 600 | 1.44 | 1.04e-81 | 65.22 (3.90) |  | |
|  | Error | 14,739 | 416 |  |  | 418.23 (4.88) |  | |
| 25°, 28° | *S* | 1 | 13,774 | 7.62 | 6.35e-3 | Fixed | 0.40 | |
|  | *T* | 1 | 2,829,916 | 733.80 | 1.09e-64 | Fixed |  | |
|  | *S*×*T* | 1 | 24,802 | 28.41 | 2.97e-7 | Fixed |  | |
|  | *L* | 185 | 11,706 | 2.44 | 1.96e-10 | 28.12 (5.34) |  | |
|  | *S*×*L* | 185 | 1,815 | 2.08 | 6.52e-7 | 7.24 (1.81) |  | |
|  | *T*×*L* | 176 | 3,865 | 4.28 | 1.52e-21 | 23.15 (3.37) |  | |
|  | *S*×*T*×*L* | 176 | 875 | 6.04 | 5.19e-117 | 12.12 (1.58) |  | |
|  | *Rep*(*T*×*L*) | 8,003 | 175 | 1.20 | 5.91e-17 | 5.35 (0.64) |  | |
|  | *S*×*Rep*(*T*×*L*) | 8,003 | 145 | 1.39 | 3.17e-81 | 14.23 (0.87) |  | |
|  | Error | 30,455 | 105 |  |  | 104.96 (0.85) |  | |
| Females  (25°, 28°) | *T* | 1 | 1,175,566 | 504.32 | 1.45e-53 | Fixed | 0.38 | |
|  | *L* | 185 | 6,400 | 2.74 | 2.00e-11 | 32.35 (5.79) |  | |
|  | *T*×*L* | 176 | 2,337 | 14.21 | 0 | 34.02 (3.93) |  | |
|  | *Rep*(*T*×*L*) | 8,003 | 166 | 1.56 | 6.32e-121 | 20.87 (1.01) |  | |
|  | Error | 15,433 | 106 |  |  | 106.12 (1.21) |  | |
| Males  (25°, 28°) | *T* | 1 | 1,673,587 | 695.83 | 4.62e-63 | Fixed | 0.42 | |
|  | *L* | 185 | 7,120 | 2.96 | 7.41e-13 | 38.69 (6.53) |  | |
|  | *T*×*L* | 176 | 2,411 | 15.64 | 0 | 36.30 (4.15) |  | |
|  | *Rep*(*T*×*L*) | 8,003 | 155 | 1.50 | 2.74e-100 | 18.29 (0.98) |  | |
|  | Error | 15,022 | 103 |  |  | 103.77 (1.20) |  | |
|  |  |  |  |  |  |  |  | |
|  |  |  |  |  |  |  |  | |
| 18°, 28° | *S* | 1 | 136,134 | 37.04 | 6.58e-9 | Fixed | 0.39 | |
|  | *T* | 1 | 32,567,651 | 1576.29 | 1.86e-89 | Fixed |  | |
|  | *S*×*T* | 1 | 155,544 | 47.26 | 1.05e-10 | Fixed |  | |
|  | *L* | 183 | 34,832 | 1.65 | 4.58e-4 | 54.84 (17.78) |  | |
|  | *S*×*L* | 183 | 3,681 | 1.12 | 0.23 | 3.31 (4.20) |  | |
|  | *T*×*L* | 175 | 20,705 | 6.08 | 6.10e-31 | 141.79 (18.28) |  | |
|  | *S*×*T*×*L* | 175 | 3,298 | 6.70 | 5.16e-135 | 44.38 (5.54) |  | |
|  | *Rep*(*T*×*L*) | 7,973 | 604 | 1.22 | 1.47e-19 | 20.73 (2.27) |  | |
|  | *S*×*Rep*(*T*×*L*) | 7,973 | 494 | 1.31 | 5.95e-55 | 39.10 (3.08) |  | |
|  | Error | 29,483 | 377 |  |  | 380.50 (3.15) |  | |
| Females  (18°, 28°) | *T* | 1 | 14,301,215 | 1179.21 | 1.11e-79 | Fixed | 0.39 | |
|  | *L* | 183 | 18,342 | 1.51 | 3.11e-3 | 48.16 (18.87) |  | |
|  | *T*×*L* | 175 | 12,155 | 22.47 | 0 | 186.07 (20.84) |  | |
|  | *Rep*(*T*×*L*) | 7,973 | 545 | 1.50 | 2.15e-98 | 61.83 (3.48) |  | |
|  | Error | 14,992 | 364 |  |  | 367.18 (4.26) |  | |
| Males  (18°, 28°) | *T* | 1 | 18,367,859 | 1553.60 | 5.73e-89 | Fixed | 0.39 | |
|  | *L* | 183 | 20,192 | 1.71 | 2.02e-4 | 67.92 (20.58) |  | |
|  | *T*×*L* | 175 | 11,855 | 21.45 | 0 | 186.31 (20.86) |  | |
|  | *Rep*(*T*×*L*) | 7,973 | 557 | 1.43 | 4.58e-75 | 57.96 (3.67) |  | |
|  | Error | 14,491 | 391 |  |  | 393.94 (4.64) |  | |
| 25° | *S* | 1 | 38,550 | 18.47 | 1.73e-5 | Fixed | 0.42 | |
|  | *L* | 185 | 10,639 | 5.24 | 9.69e-28 | 68.14 (8.92) |  | |
|  | *S*×*L* | 185 | 1,989 | 10.68 | 1.82e-233 | 28.77 (3.30) |  | |
|  | *Rep*(*L*) | 4,084 | 230 | 1.23 | 1.50e-11 | 7.82 (1.18) |  | |
|  | *S*×*Rep*(*L*) | 4,084 | 187 | 1.40 | 1.24e-44 | 18.84 (1.57) |  | |
|  | Error | 15,520 | 133 |  |  | 133.77 (1.52) |  | |
| Females (25°) | *L* | 185 | 5,959 | 28.27 | 0 | 89.63 (9.66) | 0.40 | |
|  | *Rep*(*L*) | 4,084 | 212 | 1.57 | 6.33e-65 | 27.16 (1.81) |  | |
|  | Error | 7,885 | 135 |  |  | 134.96 (2.14) |  | |
| Males (25°) | *L* | 185 | 6,669 | 32.64 | 0 | 104.26 (11.18) | 0.44 | |
|  | *Rep*(*L*) | 4,084 | 206 | 1.56 | 6.28e-62 | 26.18 (1.82) |  | |
|  | Error | 7,635 | 132 |  |  | 132.53 (2.15) |  | |
| 18° | *S* | 1 | 296,566 | 48.73 | 5.29e-11 | Fixed | 0.39 | |
|  | *L* | 182 | 49,216 | 7.80 | 7.49e-40 | 354.38 (42.82) |  | |
|  | *S*×*L* | 182 | 6,111 | 7.02 | 5.73e-138 | 84.98 (10.38) |  | |
|  | *Rep*(*L*) | 4,054 | 1,075 | 1.23 | 1.95e-11 | 38.16 (5.73) |  | |
|  | *S*×*Rep*(*L*) | 4,054 | 874 | 1.27 | 7.99e-23 | 67.26 (7.78) |  | |
|  | Error | 14,548 | 687 |  |  | 689.24 (8.07) |  | |
| Females (18°) | *L* | 182 | 27,037 | 28.42 | 0 | 420.68 (45.67) | 0.39 | |
|  | *Rep*(*L*) | 4,054 | 960 | 1.46 | 6.55e-45 | 107.27 (8.70) |  | |
|  | Error | 7,444 | 656 |  |  | 659.00 (10.81) |  | |
| Males (18°) | *L* | 182 | 28,364 | 28.69 | 0 | 457.87 (49.71) | 0.39 | |
|  | *Rep*(*L*) | 4,054 | 997 | 1.38 | 1.28e-32 | 103.68 (9.38) |  | |
|  | Error | 7,104 | 720 |  |  | 720.28 (12.03) |  | |
| 28° | *S* | 1 | 625 | 0.91 | 0.34 | Fixed | 0.36 | |
|  | *L* | 176 | 4,958 | 7.06 | 5.10e-35 | 33.17 (4.16) |  | |
|  | *S*×*L* | 176 | 687 | 6.75 | 1.78e-126 | 9.12 (1.14) |  | |
|  | *Rep*(*L*) | 3,919 | 118 | 1.15 | 5.71e-6 | 2.79 (0.62) |  | |
|  | *S*×*Rep*(*L*) | 3,919 | 102 | 1.37 | 7.12e-37 | 9.42 (0.87) |  | |
|  | Error | 14,935 | 75 |  |  | 75.01 (0.87) |  | |
| Females (28°) | *L* | 176 | 2,788 | 24.01 | 0 | 41.05 (4.56) | 0.35 | |
|  | *Rep*(*L*) | 3,919 | 117 | 1.54 | 6.95e-57 | 14.31 (1.03) |  | |
|  | Error | 7,548 | 76 |  |  | 76.03 (1.24) |  | |
| Males (28°) | *L* | 176 | 2,870 | 28.14 | 0 | 43.57 (4.81) | 0.37 | |
|  | *Rep*(*L*) | 3,919 | 103 | 1.39 | 1.60e-33 | 10.10 (0.93) |  | |
|  | Error | 7,387 | 74 |  |  | 73.97 (1.22) |  | |
