# Supplementary material for: Context-dependent genetic architecture of Drosophila life span
Source: PLoS Biol. 2020 Mar 5;18(3):e3000645. doi: 10.1371/journal.pbio.3000645 (PMC7077879; doi:10.1371/journal.pbio.3000645)
Supplement: S3 Table — Sex, temperature, and their interaction are fixed effects; the rest are random. Full mixed model, factorial ANOVAs as well as reduced models by temperature and sex are given. σ2, REML variance component estimate; df, degrees of freedom; F, F-ratio test; H2, broad-sense heritability; L, DGRP Line; MS, Type III mean squares; Rep, Replicate vial; S, Sex; SE, standard error; T, Temperature. (DOCX) [file pbio.3000645.s003.docx]

| **Analysis** | **Source** | **df** | **MS** | **F** | ***P*** | ***σ*^2^ (SE)** | ***H*^2^** |  |
| --- | --- | --- | --- | --- | --- | --- | --- | --- |
| Full Model | *S* | 1 | 0.0054 | 0.07 | 7.97e-1 | Fixed | 0.71 |  |
|  | *T* | 2 | 235.4203 | 1247.77 | 4.27e-162 | Fixed |  |  |
|  | *S*×*T* | 2 | 0.3801 | 6.70 | 1.38e-3 | Fixed |  |  |
|  | *L* | 185 | 0.4931 | 2.31 | 1.72e-7 | 0.0240 (0.0047) |  |  |
|  | *S*×*L* | 185 | 15.0777 | 1.44 | 4.88e-3 | 0.0042 (0.0016) |  | |
|  | *T*×*L* | 358 | 67.5448 | 3.33 | 1.97e-19 | 0.0333 (0.0037) |  | |
|  | *S*×*T*×*L* | 358 | 0.0567 | 1.89 | 7.25e-10 | 0.0134 (0.0022) |  | |
|  | Error | 1,092 | 0.0300 |  |  | 0.0300 (0.0013) |  | |
| Females (all temperatures) | *T* | 2 | 108.4116 | 3601.90 | 0 | Fixed | 0.72 | |
|  | *L* | 185 | 0.3021 | 9.95 | 2.95e-8 | 0.0310 (0.0057) |  | |
|  | *T*×*L* | 358 | 0.1206 | 3.97 | 7.60e-23 | 0.0456 (0.0047) |  | |
|  | Error | 546 | 0.0304 |  |  | 0.0304 (0.0018) |  | |
| Males (all temperatures) | *T* | 2 | 127.7965 | 4319.06 | 0 | Fixed | 0.71 | |
|  | *L* | 185 | 0.2725 | 9.21 | 5.06e-7 | 0.0251 (0.0051) |  | |
|  | *T*×*L* | 358 | 0.1248 | 4.22 | 6.64e-24 | 0.0478 (0.0048) |  | |
|  | Error | 546 | 0.0296 |  |  | 0.0296 (0.0018) |  | |
| 18°, 25° | *S* | 1 | 0.1325 | 4.26 | 3.94e-2 | Fixed | 0.70 | |
|  | *T* | 1 | 237.03 | 7,616.99 | 0 | Fixed |  | |
|  | *S*×*T* | 1 | 0.3993 | 12.83 | 3.63e-4 | Fixed |  | |
|  | *L* | 185 | 0.3876 | 12.46 | 9.58e-6 | 0.0243 (0.0057) |  | |
|  | *S*×*L* | 185 | 0.0756 | 2.43 | 1.83e-2 | 0.0051 (0.0024) |  | |
|  | *T*×*L* | 182 | 0.1753 | 5.63 | 2.05e-10 | 0.0300 (0.0048) |  | |
|  | *S*×*T*×*L* | 182 | 0.0549 | 1.76 | 3.26e-5 | 0.0120 (0.0030) |  | |
|  | Error | 738 | 0.0311 |  |  | 0.0311 (0.0016) |  | |
| Females  (18°, 25°) | *T* | 1 | 108.99 | 3498.05 | 2.42e-190 | Fixed | 0.70 | |
|  | *L* | 185 | 0.2520 | 8.09 | 1.44e-7 | 0.0367 (0.0072) |  | |
|  | *T*×*L* | 182 | 0.1066 | 3.42 | 1.75e-11 | 0.0376 (0.0057) |  | |
|  | Error | 369 | 0.0312 |  |  | 0.0311 (0.0023) |  | |
| Males  (18°, 25°) | *T* | 1 | 128.44 | 4132.55 | 1.62e-202 | Fixed | 0.69 | |
|  | *L* | 185 | 0.2112 | 6.80 | 2.43e-4 | 0.0223 (0.0064) |  | |
|  | *T*×*L* | 182 | 0.1236 | 3.98 | 9.19e-13 | 0.0460 (0.0065) |  | |
|  | Error | 369 | 0.0311 |  |  | 0.0311 (0.0023) |  | |
| 25°, 28° | *S* | 1 | 0.1854 | 5.87 | 1.57e-2 | Fixed | 0.70 | |
|  | *T* | 1 | 33.2762 | 1052.92 | 1.98e-143 | Fixed |  | |
|  | *S*×*T* | 1 | 0.0262 | 0.83 | 3.63e-1 | Fixed |  | |
|  | *L* | 185 | 0.4422 | 13.99 | 1.63e-8 | 0.0352 (0.0064) |  | |
|  | *S*×*L* | 185 | 0.0734 | 2.32 | 1.62e-2 | 0.0052 (0.0024) |  | |
|  | *T*×*L* | 176 | 0.1465 | 4.63 | 8.24e-9 | 0.0235 (0.0042) |  | |
|  | *S*×*T*×*L* | 176 | 0.0527 | 1.67 | 1.42e-4 | 0.0106 (0.0029) |  | |
|  | Error | 726 | 0.0316 |  |  | 0.0316 (0.0016) |  | |
| Females  (25°, 28°) | *T* | 1 | 15.7180 | 510.09 | 3.61e-71 | Fixed | 0.71 | |
|  | *L* | 185 | 0.2514 | 8.16 | 2.84e-7 | 0.0371 (0.0074) |  | |
|  | *T*×*L* | 176 | 0.1055 | 3.42 | 3.95e-11 | 0.0379 (0.0058) |  | |
|  | Error | 363 | 0.0308 |  |  | 0.0308 (0.0023) |  | |
| Males  (25°, 28°) | *T* | 1 | 17.5843 | 542.84 | 4.47e-74 | Fixed | 0.70 | |
|  | *L* | 185 | 0.2641 | 8.15 | 7.41e-13 | 0.0437 (0.0075) |  | |
|  | *T*×*L* | 176 | 0.0937 | 2.89 | 0 | 0.0305 (0.0051) |  | |
|  | Error | 363 | 0.0324 |  |  | 0.0324 (0.0024) |  | |
| 18°, 28° | *S* | 1 | 0.0360 | 1.33 | 2.50e-1 | Fixed | 0.74 | |
|  | *T* | 1 | 433.4751 | 15,946.3 | 0 | Fixed |  | |
|  | *S*×*T* | 1 | 0.6362 | 23.40 | 1.61e-6 | Fixed |  | |
|  | *L* | 183 | 0.3456 | 12.71 | 8.38e-2 | 0.0085 (0.0061) |  | |
|  | *S*×*L* | 183 | 0.0703 | 2.59 | 2.04e-1 | 0.0021 (0.0025) |  | |
|  | *T*×*L* | 175 | 0.2463 | 9.06 | 8.82e-12 | 0.0492 (0.0073) |  | |
|  | *S*×*T*×*L* | 175 | 0.0632 | 2.33 | 8.62e-8 | 0.0177 (0.0034) |  | |
|  | Error | 720 | 0.0272 |  |  | 0.0272 (0.0014) |  | |
| Females  (18°, 28°) | *T* | 1 | 200.4488 | 6,880.05 | 4.70e-238 | Fixed | 0.73 | |
|  | *L* | 183 | 0.2218 | 7.61 | 2.91e-2 | 0.0144 (0.0076) |  | |
|  | *T*×*L* | 175 | 0.1508 | 5.18 | 8.31e-14 | 0.0648 (0.0088) |  | |
|  | Error | 360 | 0.0291 |  |  | 0.0291 (0.0022) |  | |
| Males  (18°, 28°) | *T* | 1 | 233.6625 | 9,260.52 | 6.17e-259 | Fixed | 0.75 | |
|  | *L* | 183 | 0.1940 | 7.69 | 1.68e-1 | 0.0066 (0.0069) |  | |
|  | *T*×*L* | 175 | 0.1587 | 6.29 | 3.84e-15 | 0.0691 (0.0089) |  | |
|  | Error | 360 | 0.0252 |  |  | 0.0252 (0.0019) |  | |
| 25° | *S* | 1 | 0.0348 | 0.98 | 3.22e-1 | Fixed | 0.66 | |
|  | *L* | 185 | 0.2834 | 8.01 | 1.03e-13 | 0.0553 (0.0075) |  | |
|  | *S*×*L* | 185 | 0.0619 | 1.75 | 6.77e-5 | 0.0132 (0.0035) |  | |
|  | Error | 372 | 0.0354 |  |  | 0.0354 (0.0026) |  | |
| Females (25°) | *L* | 185 | 0.1733 | 5.29 | 9.07e-15 | 0.0702 (0.0092) | 0.68 | |
|  | Error | 186 | 0.0328 |  |  | 0.0328 (0.0034) |  | |
| Males (25°) | *L* | 185 | 0.1720 | 4.52 | 0 | 0.0670 (0.0092) | 0.64 | |
|  | Error | 186 | 0.0380 |  |  | 0.0380 (0.0039) |  | |
| 18° | *S* | 1 | 0.4970 | 18.57 | 2.11e-5 | Fixed | 0.73 | |
|  | *L* | 182 | 0.2813 | 10.51 | 1.30e-12 | 0.0531 (0.0076) |  | |
|  | *S*×*L* | 182 | 0.0688 | 2.57 | 9.39e-9 | 0.0210 (0.0037) |  | |
|  | Error | 366 | 0.0268 |  |  | 0.0268 (0.0020) |  | |
| Females (18°) | *L* | 182 | 0.1867 | 6.33 | 1.06e-15 | 0.0786 (0.0099) | 0.73 | |
|  | Error | 183 | 0.0295 |  |  | 0.0295 (0.0031) |  | |
| Males (18°) | *L* | 182 | 0.1634 | 6.80 | 4.10e-16 | 0.0697 (0.0086) | 0.74 | |
|  | Error | 183 | 0.0240 |  |  | 0.0240 (0.0025) |  | |
| 28° | *S* | 1 | 0.1869 | 6.77 | 9.66e-3 | Fixed | 0.74 | |
|  | *L* | 176 | 0.3133 | 11.35 | 1.57e-13 | 0.0622 (0.0085) |  | |
|  | *S*×*L* | 176 | 0.0648 | 2.34 | 1.29e-7 | 0.0186 (0.0036) |  | |
|  | Error | 354 | 0.0276 |  |  | 0.0276 (0.0021) |  | |
| Females (28°) | *L* | 176 | 0.1876 | 6.53 | 2.03e-15 | 0.0794 (0.0101) | 0.73 | |
|  | Error | 177 | 0.0288 |  |  | 0.0288 (0.0030) |  | |
| Males (28°) | *L* | 176 | 0.1905 | 7.19 | 6.21e-16 | 0.0820 (0.0102) | 0.76 | |
|  | Error | 177 | 0.0264 |  |  | 0.0265 (0.0028) |  | |
