# Supplementary figures and images for: Context-dependent genetic architecture of Drosophila life span
Source: PLoS Biol. 2020 Mar 5;18(3):e3000645. doi: 10.1371/journal.pbio.3000645 (PMC7077879; doi:10.1371/journal.pbio.3000645)

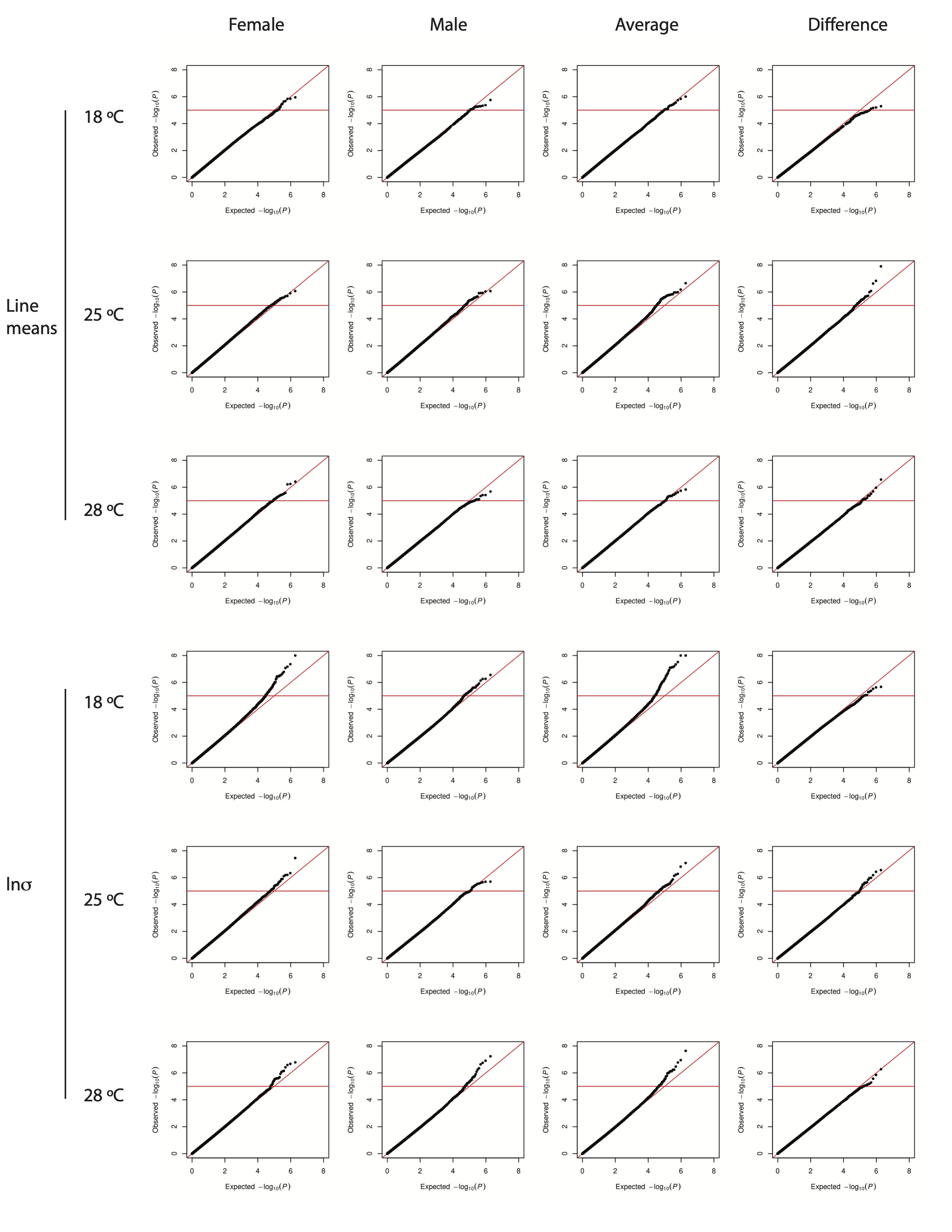

Supplement: S1 Fig — QQ plots of P values where the x axis is the expected P value based on a uniform distribution while the y axis is the observed P value. The horizontal line indicates the 10−5 cutoff chosen to declare significance. Code to generate the Q-Q plots is available at https://github.com/qgg-lab/dgrp-lifespan/. (TIFF) [file pbio.3000645.s011.tiff]

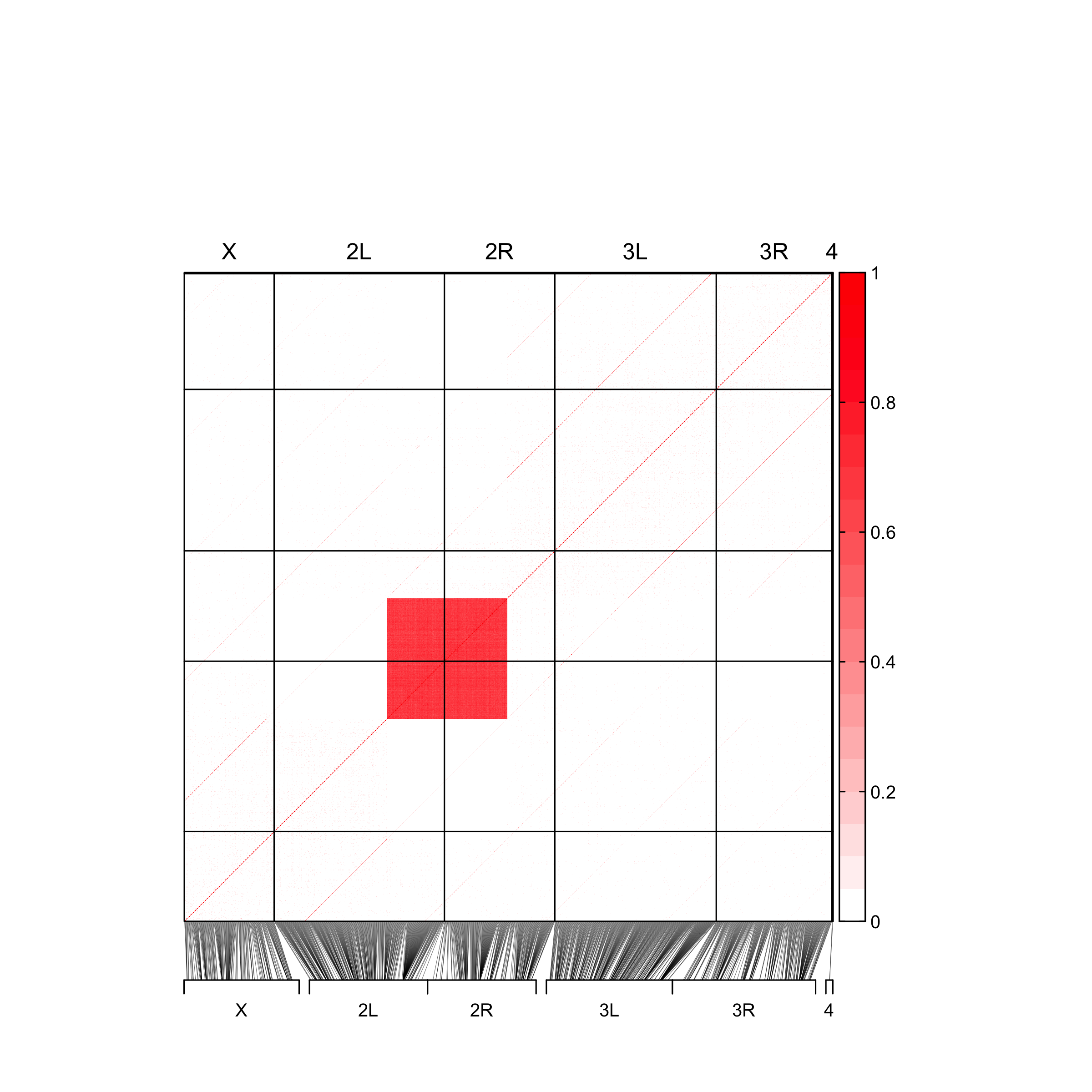

Supplement: S2 Fig — Heatmap showing the pairwise r2 between significant variants in the DGRP. Below the heatmap, the relative positions between the variants are indicated on the chromosomes by lines connecting the positions on the heatmap and positions on the chromosomes. The raw data for the information depicted in this figure are available at https://github.com/qgg-lab/dgrp-lifespan/. (TIFF) [file pbio.3000645.s012.tiff]

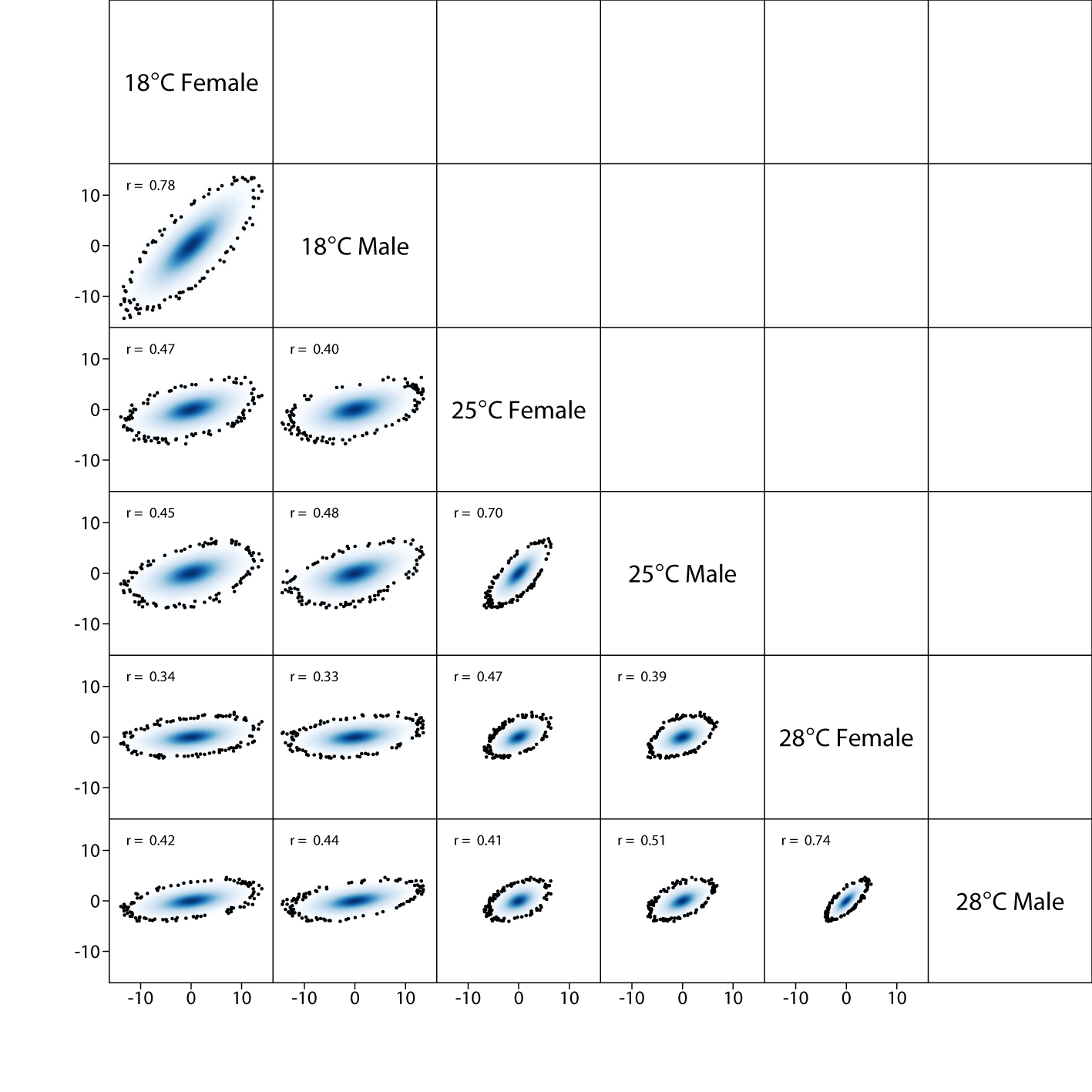

Supplement: S3 Fig — Estimated allelic effects (for mean life span) in each environment are plotted against each other where the x axis is the effect in the environment indicated on the top of the column of cells (along the diagonal) and the y axis is the effect in the environment indicated on the right of the row of cells (along the diagonal). The data are plotted as a smoothed two-dimensional density plot with large effects (low-density areas) plotted as points on the edge. The darkness of the color indicates density of points. Spearman’s correlation is also indicated on the top left corner of the plot. The raw data for the information depicted in this figure are available at https://github.com/qgg-lab/dgrp-lifespan/. (TIF) [file pbio.3000645.s013.tif]

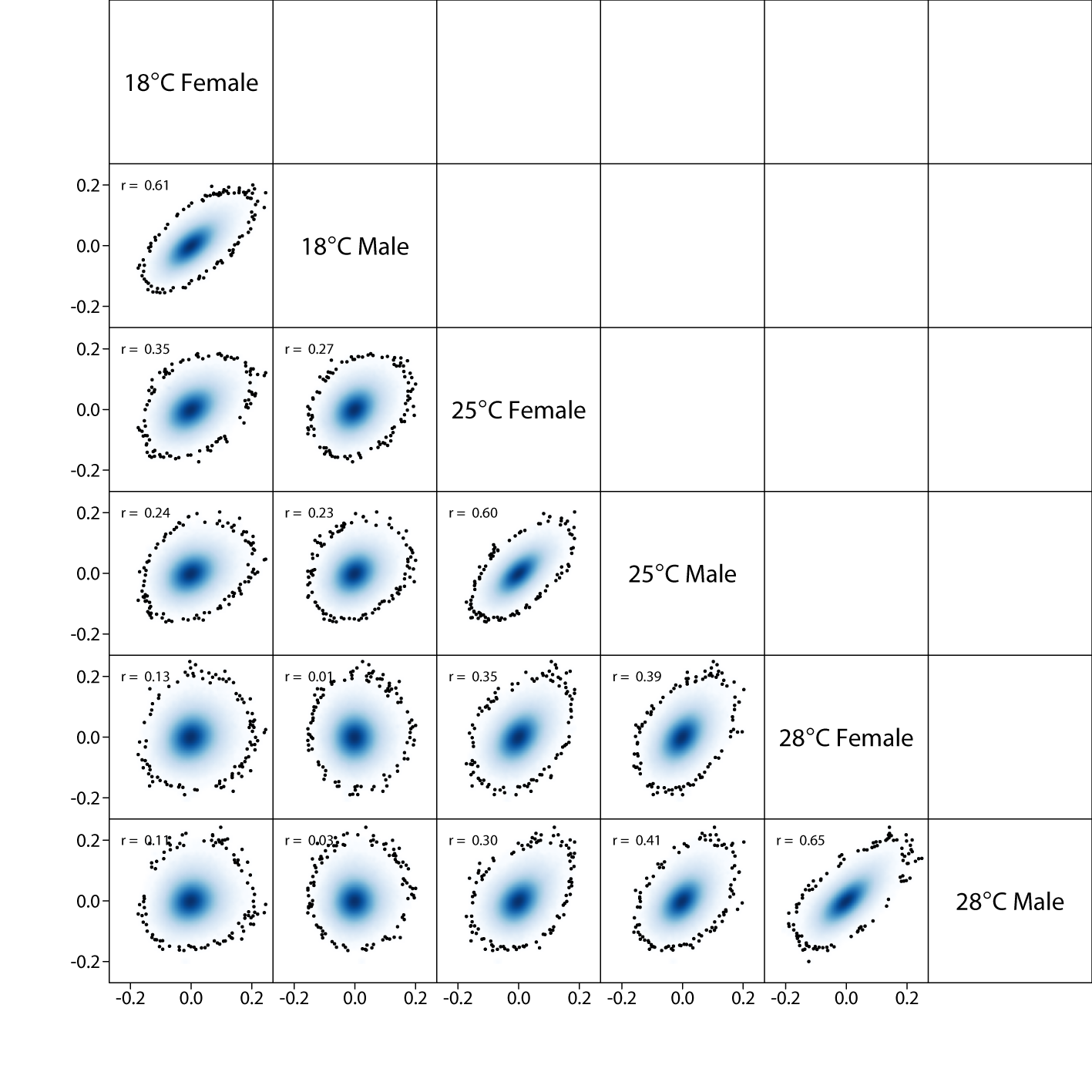

Supplement: S4 Fig — Estimated allelic effects (for lnσε life span) in each environment are plotted against each other where the x axis is the effect in the environment indicated on the top of the column of cells (along the diagonal) and the y axis is the effect in the environment indicated on the right of the row of cells (along the diagonal). The data are plotted as a smoothed two-dimensional density plot with large effects (low-density areas) plotted as points on the edge. The darkness of the color indicates density of points. Spearman’s correlation is also indicated on the top left corner of the plot. The raw data for the information depicted in this figure are available at https://github.com/qgg-lab/dgrp-lifespan/. (TIF) [file pbio.3000645.s014.tif]

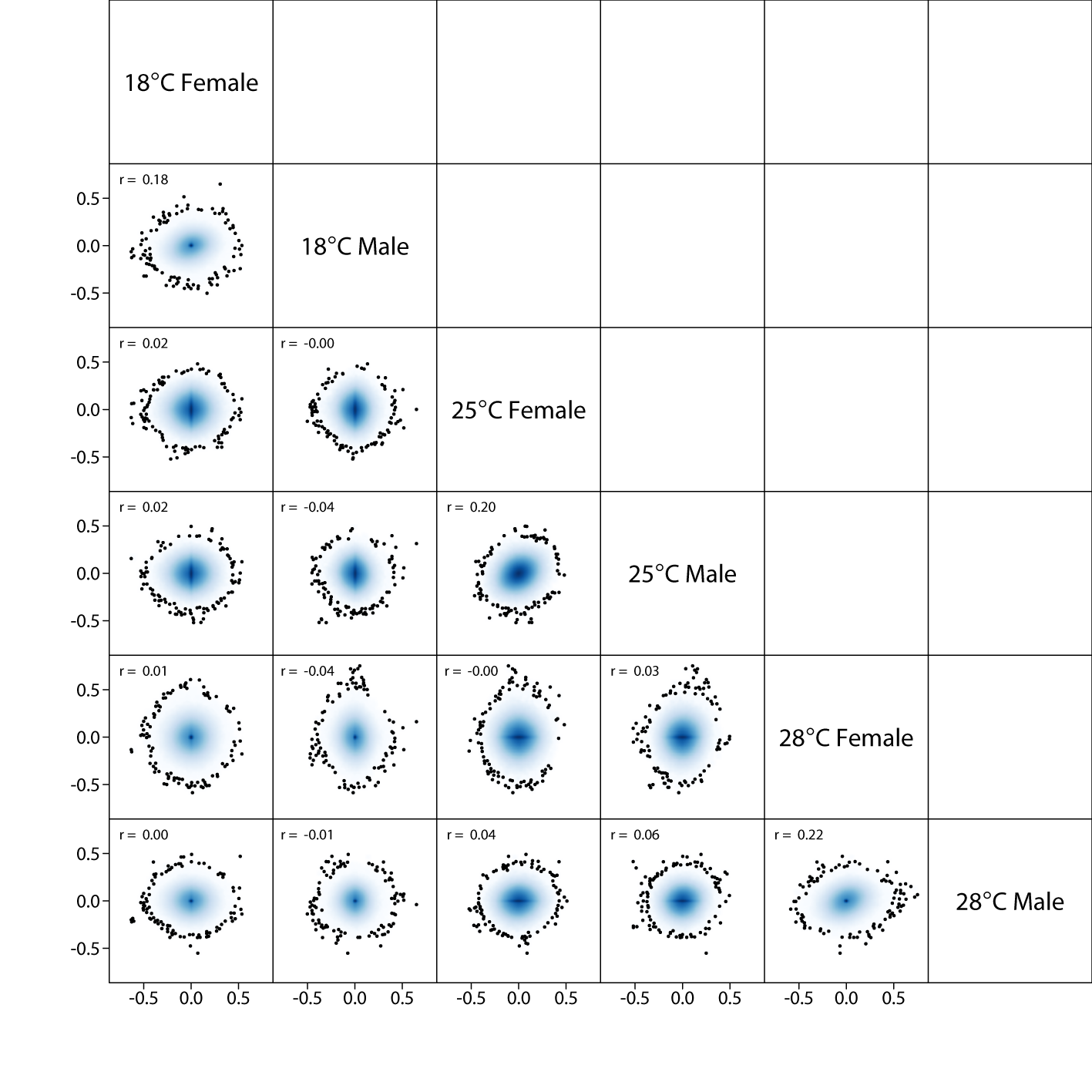

Supplement: S5 Fig — Estimated allelic effects (allele frequency difference between the long-living and random pools) in each environment are plotted against each other where the x axis is the effect in the environment indicated on the top of the column of cells (along the diagonal) and the y axis is the effect in the environment indicated on the right of the row of cells (along the diagonal). The data are plotted as a smoothed two-dimensional density plot with large effects (low-density areas) plotted as points on the edge. The darkness of the color indicates density of points. Spearman’s correlation is also indicated on the top left corner of the plot. The raw data for the information depicted in this figure are available at https://github.com/qgg-lab/dgrp-lifespan/. (TIF) [file pbio.3000645.s015.tif]
